# Solid Boundary Output Feedback Control of the Stefan Problem: The Enthalpy Approach

**DOI:** 10.1109/tac.2022.3197704

**Published:** 2022-08-09

**Authors:** Bryan Petrus, Zhelin Chen, Hamza El-Kebir, Joseph Bentsman, Brian G. Thomas

**Affiliations:** University of Illinois Urbana-Champaign, Champaign, IL 61801 USA. He is now with Nucor Steel Decatur, Decatur, AL 35673 USA; University of Illinois Urbana-Champaign, Champaign, IL 61801 USA; University of Illinois Urbana-Champaign, Champaign, IL 61801 USA; University of Illinois Urbana-Champaign, Champaign, IL 61801 USA; Colorado School of Mines, Golden, CO 80401 USA

**Keywords:** Control, enthalpy, nonlinear partial differential equations, solidification, Stefan problem

## Abstract

By taking enthalpy—an internal energy of a diffusion-type system—as the system state and expressing it in terms of the temperature profile and the phase-change interface position, the output feedback boundary control laws for a fundamentally nonlinear single-phase one-dimensional (1-D) PDE process model with moving boundaries, referred to as the Stefan problem, are developed. The control objective is tracking of the spatiotemporal temperature and temporal interface (solidification front) trajectory generated by the reference model. The external boundaries through which temperature sensing and heat flux actuation are performed are assumed to be solid. First, a full-state single-sided tracking feedback controller is presented. Then, an observer is proposed and proven to provide a stable full-state reconstruction. Finally, by combining a full-state controller with an observer, the output feedback trajectory tracking control laws are presented and the closed-loop convergence of the temperature and the interface errors proven for the single-sided and the two-sided Stefan problems. Simulation shows the exponential-like trajectory convergence attained by the implementable smooth bounded control signals.

## I. Introduction

PHASE-CHANGE phenomena permeate the physical world and underpin the brains and the brawn of modern civilization through, respectively, semiconductor manufacturing [1] and steel casting [2]. One of the most widely encountered such phenomena is the thermally induced phase transition typically modeled as a Stefan partial-differential-equation/ordinary-differential-equation (PDE/ODE) system, also referred to as the Stefan problem [3]. In the latter system, the moving boundary separates the material into two subdomains with different phases, and the temperature inside each subdomain is typically modeled by a linear diffusion equation. The speed of the moving boundary is proportional to the difference between the heat fluxes on the two sides of the boundary, balanced by the materialdependent energy of phase transition (enthalpy jump) between them. The latter relation is represented by an ODE referred to as the Stefan condition. The discontinuous temperature gradients at the boundary make the problem fundamentally nonlinear. The Stefan description has been employed in a number of areas, including electrical discharge machining [4], additive manufacturing [5], and very recently electrosurgery [6], [7]. This growing technological importance brings out the need for observation and control of such systems.

The earlier work on Stefan problem control can be generally divided into the following three categories: numerical optimization methods [8], [9], solutions of the inverse Stefan problem [10], [11], [12], and feedback control methods [13], [14], [15]. The numerical optimization methods in [8] and [9] can take into account realistic metallurgical constraints and quality requirements. However, since the simulation involved is highly complex and nonlinear, they cannot realistically run in real time. The inverse methods [10], [11], [12] and feedback control methods [13], [14], [15] use the Stefan problem as a model and focus on control of the boundary position. The inverse problem, as solved in [11] directly and in [10] and [12] by minimizing a cost functional, is very numerically complex and, thus, limited to the design of the open-loop control schemes. The feedback control methods are better suited for real-time control, but have their drawbacks. The control in [13] and [14] is simplified to the thermostat-style one, allowing rigorous performance analysis. In [15], PI controllers are designed based on a discretized form of the Stefan problem, applicable directly to the solidification processes, but with no guaranteed performance. Recent papers [16], [17] formulated the port Hamiltonian representation of the two-phase Stefan problem with the interface dynamics included, however, the boundary controller development using the newly proposed setting is still an ongoing research.

The advent of thin slab casting pioneered by Nucor Crawfordsville (USA) in 1989 [18] re-energized the Stefan problem controller development, since the steel solidification process could no longer be properly managed by a human operator. The Stefan problem control in the latter case is especially challenging, since only the solid boundary sensing and actuation are available and control of the surface temperature as well the internal spatially varying temperature profile of the solidifying shell thickness is desirable to ensure safety and maximize the steel quality.

The controller development for the latter system by the present authors started by employing the one-dimensional (1-D) Stefan-like through-thickness solidification model proposed in [19]. Aggregating 200 such models, referred to as *slices*, along the caster and using the outflow cooling mold water temperature as the sensed variable, [20], [21] obtained a real-time two-dimensional (2-D) solidifying steel shell thickness and temperature profile estimator and used it in the inferential closed-loop shell surface temperature “setline” (the string of setpoints along the caster) tracking through a PID controller bank with anti-windup. The two-sided controller was implemented on a thin slab caster at Nucor Steel Decatur in 2006. Shortly thereafter, the real-time readout from the two-sided estimator in the open-loop permitted an operator to prevent the solidification escape (“whale”), putting the estimator into permanent use ever since. Several successful tests of the closed-loop system were also performed, each up to several hours in length [21], giving the first industrial real-time inferentially closed-loop control with the Stefan-type model based software sensor, described in [22] (patent [23]). The first author of [20] and coworkers subsequently implemented similar well-calibrated controllers at seven ArcelorMittal plants [24].

**Fig. 1.**
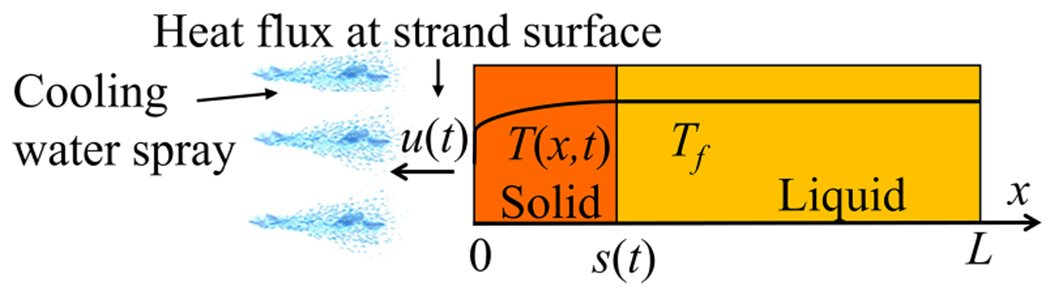
Schematic of one-dimensional (1-D) Stefan problem.

However, the increased production requirements for crack avoidance, bulging minimization, and full steel solidification at the caster exit cannot be simultaneously addressed by the current Nucor Steel production control system [22]. This article is part of a comprehensive effort toward control system upgrades to meet these demands. This article also provides guidance for the control-oriented continuous caster equipment upgrades, showing what is fundamentally achievable if the corresponding sensors and actuators are put into place. In a departure from [20], [21], and [22], the control objective then becomes the simultaneous control of the steel shell temperature profile and thickness. This objective is addressed through replacing the PID framework of [20], [21], and [22] by the reference model setting introduced in [25], which involves the reference model path planning [10], the online plant model recalibration [26], and the suitable control law for tracking the desired reference model trajectory, developed in this article. Petrus et al. [26] give the system layout (see Fig. 1) and the modeling framework (see Section II) adhered to in this article.

The first reference model-based boundary feedback controller using the two-phase Stefan steel solidification model proposed in [25] produced, however, irregular control signals due to the choice of temperature as the system state and the variable to control. Faced with this difficulty, Petrus et al. [27] proposed taking the *enthalpy* (the internal system energy), rather than the temperature, as the system state, which along with the temperature includes the phase-change interface position. The implication for the full-state feedback control law was the inclusion into it, along with the *spatially integrated temperature error* over the actuated spatial domain, the phase-change *interface position error*, further referred to as SITE/IPE form. The latter accounted for the full system internal energy change. The implication for the observer design was the inclusion into an observer of the Stefan condition—the ODE governing the interface velocity, with the surface temperature measurement injection into it. The resulting control and PDE-based estimation algorithms admitted a rigorous stability proof for the full-state feedback and yielded the estimation and the output feedback control laws for a single-sided single-phase Stefan problem with smooth control signals and exponential convergence rate seen in simulations, but the convergence of the estimator and the output feedback errors was not proven. The follow-up paper [28] extended the full-state feedback result of [27] to a two-sided Stefan problem to match the actual continuous casters dynamics, but no output feedback was provided.

For distributed parameter diffusion-type thermal systems, common Lyapunov functionals involve the $L_{2}$ temperature norm and the Sobolev norms that include the $L_{2}$ norms of the temperature and one or more of its spatial derivatives. These norms generate Hilbert spaces, which allow for proving boundedness and well-posedness. However, in the systems with heat transfer, temperature is more properly thought of as the system output, rather than the state. The actual internal energy in heat diffusion is called the enthalpy [29]. For the single-phase systems, the enthalpy is simply the temperature multiplied by a constant specific heat, making the mathematical norms of the temperature and the enthalpy equivalent. In the multiphase systems, however, the specific heat is not constant, and so the norms are no longer equivalent. In [30], an enthalpy-type quantity, referred to as entropy, was applied to stability analysis of parabolic PDE systems with fixed boundary using Sobolev embeddings. However, in no work on control of Stefan problem prior to the conference publication [27] of the present authors had enthalpy been used, permitting for the first time to attain the *simultaneous control of the spatially varying temperature profile*, distributed throughout the material, and the *solidification front*.

The approach pioneered in [25], [27], and [28] launched a series of subsequent works [31], [32], [33], [34], [35]. In these works, the Stefan problem closed-loop boundary controllers were designed using the backstepping techniques, while adopting the system-theoretic tools introduced by the enthalpy approach of [27]. The latter included the system state choice, the state-feedback SITE/IPE controller form, the nonlinear observers based on the process model with various scenarios of measurement injection into the Stefan condition ODE, and the output feedback controller structure. However, the focus of these works has been on the Stefan problem control *through the moving boundary*, opening up the applications of the Stefan problem control to a range of modern technical problems, including three-dimensional (3-D) printing via screw extrusion and laser sintering, summarized in [35].

Specifically, in [31], the authors proposed a full-state feedback control law, an observer design, and the associated output feedback control law under both Neumann and Dirichlet boundary actuation for a one-phase Stefan problem. In [34], a controller was designed for the single-sided two-phase Stefan problem that ensures, respectively, the exponential convergence of the distributed temperature profile to the spatially invariant temperature setpoint, and the boundary interface to the fixed setpoint position. However, the proposed control laws are insufficient for a solidification process like continuous steel casting, which requires a sufficiently close tracking of the spatiotemporally varying reference temperature profile and temporally varying interface position, instead of a single point, under the solid boundary sensing and actuation. The observer designed in [31] requires the measurements of the moving boundary location and its derivative, which are not applicable for the continuous casting process, where only surface temperature measurement is available. In [32], a similar observer and an output control law are proposed for the sea-ice melting problem. The proposed observer also requires the measurements of the moving boundary. In [35], Sec. 3.5], a modification of the solid boundary observer of [27] is proposed, which includes additional injection terms in the temperature equation and the boundary flux estimate, while keeping the same Stefan condition boundary temperature injection, but also as a conjecture, with no estimation error convergence proof presented. This observer also does not fit the output feedback considered in this article, where the boundary flux estimate is known and equal to the control input.

Further on, in [35], Sec. 6.2], the model reference approach to trajectory tracking introduced in [26] is presented as the future challenge to solve, and it is indicated that the backstepping approach is at present inapplicable to this problem due to the difficulty in generating the backstepping transformation. This article, however, gives the convergence proofs for both the observer and the output feedback temperature and interface position errors in trajectory tracking, fundamentally advancing the state-of-the-art in Stefan problem control.

Using an approach different from that of [25], [27], [28], [31], [32], [33], [34], and [35] and referred to as geometric, Maidi and Corriou [36] proposed a full-state feedback control law that ensures the exponential temperature convergence. The proposed control law, however, requires the knowledge of the free boundary derivative, inaccessible in the problem considered here, and does not prove the free boundary convergence. Also the problem formulation in [36] takes a fixed free boundary setpoint as the reference, resulting in the temperature convergence to the melting temperature, while in the casting process, the time-varying free boundary and the spatiotemporally varying distributed temperature reference are needed to ensure the product quality and the operational safety. Thus, the results of [36] do not apply to the problem considered in this article.

Incorporating the conference papers [25], [27], [28] of the authors as part of this article, this article refines the proofs and fundamentally advances the results of the latter papers by the following:

1. proving convergence of the solid phase temperature profile and the solidification front estimates to the actual temperature profile and solidification front;
2. proving convergence of the closed-loop system temperature profile and solidification front to those of the reference trajectory for the output feedback controller of a single-sided single-phase Stefan problem, and showing in simulation that the convergence of both the solid phase temperature profile and the solidification front has an exponential form;
3. extending the state feedback controller for a two-sided single-phase Stefan problem to the output feedback by proving convergence of the temperature profile and the solidification front to those of the reference trajectory, and supporting this result by simulation showing the exponential-like convergence.

This article has the following structure. Section II introduces the concept of enthalpy and presents the full-state feedback controller synthesis for the single-sided Stefan problem, showing attainment of a smooth control signal and visible exponential convergence and paving the way for the controller enhancements in the subsequent sections. Section III presents the observation and the output feedback control laws, including the temperature error and solidification front convergence proofs. Section IV presents the output feedback controller for the two-sided Stefan problem and provides details of the potential implementation of the approach proposed in a continuous caster. Finally, Section V concludes this article.

## II. Control Design Based on Enthalpy

### A. System Dynamics and Control Objective

Denote the temperature of a 1-D slice of a solidifying material within as T(x,t), with 0≤x≤ℓ and t≥0, where x=0 and x=l correspond to the outer (solid) surface and the (liquid) center, respectively. The control objective will be to match a reference profile, comprising the temperature and the solidification front profiles, that meets quality-based criteria. Determining a suitable reference profile is typically carried out through experience and offline optimization, as, for example, presented by the authors in [10] and [37]. This task requires simultaneous consideration of heat transfer, mechanics, chemistry, and liquid pockets elimination equipment placement. Henceforth, we assume that a suitable profile has been provided, and the goal of the controller, then, is to match this reference profile in response to changes in initial conditions due to variations before control takes effect.

When solidification is present, the temperature of the material evolves similarly to the classical (linear parabolic) heat equation, in which heat diffuses through the material based on the Fourier’s law. In the linear heat equation, the material absorbs thermal energy proportionally to its constant specific heat. When solidifying, in contrast, the material stores an additional amount of energy called the latent heat. A simple PDE describing this behavior is known as the Stefan problem, which allows the temperature gradient to be discontinuous at a moving boundary between liquid and solid phases. The Stefan problem uses an additional boundary differential equation at this solidification front to enforce the energy balance [3].

Denote the position of the boundary between the solid and the liquid phases as s(t). Then, the following coupled PDE/ODE system models the temperature and the solidification front evolution within the slice:

(1) $$\begin{array}{cc} T_{t}(x,t)=aT_{xx}(x,t), & x\in (0,s(t))\cup (s(t),\ell ) \end{array}$$

(2) $$T(s(t),t)=T_{f},\;T_{x}(0,t)=u(t),\;T_{x}(\ell ,t)=0$$

(3) $$T(x,0)=T_{0}(x)$$

(4) $$\dot{s}(t)=b\left(T_{x}\left(s^{-}(t),t\right)-T_{x}\left(s^{+}(t),t\right)\right),\;s(0)=s_{0}.$$

In physical terms, $T_{f}$ is the melting temperature, a is the thermal diffusivity, and $b=k/\rho L_{f}$, where k is the thermal conductivity, ρ is the density, and $L_{f}$ is the latent heat. All of these physical quantities are strictly positive. The control input u is applied at the solid boundary as Neumann boundary condition. It is directly proportional to the heat flux removed from the material at the surface. Fig. 1 shows the schematic.

In most actual applications involving solidification, the temperature difference in the liquid is negligible compared to that in the solid. Therefore, neglecting the temperature gradients in the liquid is a common simplification in modeling of the continuous casters. Since this limits the temperature transients to the solid area, this simplification is sometimes called the “single-phase” Stefan problem. Then, the following simplifying but practical assumptions are made.

1. The initial conditions satisfy: $0<s_{0}<\ell ,T_{0}(x)<T_{f}$ and is nondecreasing for all $0\leq x<s_{0}$ as x increases from 0 to $s_{0}$, and $T_{0}(x)=T_{f}$ for all $x\geq s_{0}$, and are piece-wise smooth.
2. $T_{0}(x)$ is continuous on the interval [0,ℓ] and infinitely differentiable inside, except at $s_{0}$.
3. ${inf}_{t}\;u(t)\geq 0$ and ${sup}_{t}\;u(t)<\infty$.

Assumption (A3) is physically sensible, but typically not known *a priori*. However, under the control law developed mentioned below, it will be shown that this is guaranteed for simple bounds on the initial error.

The existence and uniqueness of solution to the Stefan problem (1)–(4) under assumption (A1)–(A3) were developed in [37] as briefly stated as follows.

*Lemma II.1:* The solution T(x,t) of problem (1)–(4) under the assumptions (A1)–(A3) satisfies

(5) $$0\leq T_{x}\left(s^{-}(t),t\right)\leq M$$

where $M={max}_{t,x}\;\left\{max\left\{u(t),\frac{T_{f}-T_{0}(x)}{s_{0}-x}\right\}\right\}$.

*Proof:* Let us prove (5) for each fixed instant $t^{*}$. First, note that, the first inequality in (5) easily follows from the maximum principle. For each $t^{*}$, consider the function

(6) $$w(x,t)=M\left(s\left(t^{\text{*}}\right)-x\right)+T(x,t)-T_{f}.$$

For $0<t\leq t^{*},0<x<s(t)$, we then have

(7) $$w_{xx}=w_{t}$$

(8) $$w_{x}(0,t)=-M+T_{x}(0,t)=-M+u(t)\leq 0$$

(9) $$w(s(t),t)=M\left(s\left(t^{\text{*}}\right)-s(t)\right)\geq 0$$

(10) $$w(x,0)=M\left(s\left(t^{\text{*}}\right)-x\right)+T_{0}(x)-T_{f}\geq 0.$$

Hence, by the maximum principle [38] (Theorem 8, p. 389), w(x,t)>0 in its domain of definition. Since $w\left(s\left(t^{*}\right),t^{*}\right)=0$, it follows that $w_{x}\left(s^{-}\left(t^{*}\right),t^{*}\right)\leq 0$, which implies $T_{x}\left(s^{-}\left(t^{*}\right),t^{*}\right)\leq \;M$ for each $t^{*}$ and completes the proof of the Lemma. ∎

*Theorem II.1 (Existence):* Under the assumptions (A1)–(A3), there exists a solution to the problem (1)–(4), which is defined for all t>0.

*Proof:* For each θ sufficiently small, define

(11) $$T_{0}^{\theta }:=\{\begin{array}{ll} T_{0}, & 0\leq x\leq s_{0}-\theta \\ T_{f}, & s_{0}-\theta <x\leq s_{0}. \end{array}$$

Consider the time interval 0≤t≤θ and let $\left(T^{\theta },s^{\theta }\right)$ be the solution of problem (1)–(4) with $T_{0}$ replaced by $T_{0}^{\theta }$, and s(t) replaced by $s^{\theta }(t):=s_{0}$. It is shown in [39] that problem (1)–(4) has a unique solution $T^{\theta }(x,t)$ for a given real-valued function $s^{\theta }(t):=s_{0}$. In this case, it is easy to show that $T_{x}^{\theta }\left(s^{\theta -}(t),t\right):=$ $T_{x}^{\theta }\left(s_{0}^{-}(t),t\right)$ exists and is continuous in [0,θ] and by Lemma 2.1, we have $0\leq T_{x}^{\theta }\left(s^{\theta -}(t),t\right)\leq M$. In the second time interval θ≤t≤2θ, by retarding the argument in (4), we obtain

(12) $$s^{\theta }(t)=s_{0}+\int_{\theta }^{t}T_{x}^{\theta }\left(s^{\theta }(\tau -\theta ),\tau -\theta \right)d\tau .$$

Next we obtain $T^{\theta }(x,t)$ for θ≤t≤2θ by solving (1)–(4) with new initial condition $T^{\theta }(x,\theta )$ at t=θ, and boundary s given by $s^{\theta }(t)$ for θ≤t≤2θ.

Then, (12) can be used to define $s^{\theta }$ for 2θ≤t≤3θ. By induction, $\left(T^{\theta },s^{\theta }\right)$ can be defined for θ≤t≤nθ.

Then, following the proof technique of existence of solution to the two-phase Stefan problem given in [40], Th. 1], there exists solution of (1)–(4) for $0\leq t\leq t_{\delta }$, where

(13) $$t_{\delta }:=\text{inf}\left\{t^{\text{*}}:t^{\text{*}}>0,s\left(t^{\text{*}}\right)=\delta \right\},\;\delta >s_{0}.$$

Setting

(14) $$t_{0}=\underset{0\leq \delta \leq s_{0}}{\text{sup}}t_{\delta }$$

the result in Theorem 2.1 follows. ∎

*Lemma II.2 (Theorem 2 of [*40*]):* Let $\left(T_{i},s_{i}\right),i=1,2$, denote the solutions to the problem (1)–(4) for the respective boundary and initial conditions $T_{0}^{\left(i\right)},s_{0}^{\left(i\right)},u_{i},i=1,2$. Suppose $s_{0}^{\left(1\right)}\leq qs_{0}^{\left(2\right)}$, for some q>0, then there exists a constant C such that

(15) $$\frac{1}{C}\left|s_{1}(t)-s_{2}(t)\right|\leq \left|s_{0}^{\left(1\right)}-s_{0}^{\left(2\right)}\right|+\int_{0}^{s_{0}^{\left(1\right)}}\left|T_{0}^{\left(1\right)}(x)-T_{0}^{\left(2\right)}(x)\right|dx\;+\int_{s_{0}^{\left(1\right)}}^{s_{0}^{\left(2\right)}}\left|T_{0}^{\left(2\right)}(x)\right|dx+\int_{0}^{t}\left|u_{1}(\tau )-u_{2}(\tau )\right|d\tau .$$

*Proof:* Please refer to [40] for a detailed proof. ∎

*Theorem II.2 (Uniqueness):* Under assumptions (A1)–(A3), the solution to the problem (1)–(4) is unique.

*Proof:* This is an immediate corollary of Lemma 2.2 and the maximum principle [38] (Theorem 8, p. 389). ∎

### B. Reference System and Error

Assume that a known reference temperature $\overset{‾}{T}(x,t)$ and solidification front position $\overset{‾}{s}(t)$ have been determined that are the solutions to (1)–(4) under known reference control input $\overset{‾}{u}(t)$ with initial conditions $\overset{‾}{T}(x,t)={\overset{‾}{T}}_{0}$ and $\overset{‾}{s}(t)={\overset{‾}{s}}_{0}$ satisfying assumptions (A1) and (A2). One more assumption on the reference profile is required.

$\left(A4\right)\overset{\cdot }{\bar{s}}\left(t\right)>0$ for all t>0.

Denote the reference errors as $\tilde{T}(x,t)=T(x,t)-\overset{‾}{T}(x,t)$ and $\tilde{s}(t)=s(t)-\overset{‾}{s}(t)$. Also, denote the addition to the boundary heat flux as $\tilde{u}(t)=u(t)-\overset{‾}{u}(t)$. Since the solutions to (1)–(4) are continuous everywhere on the spatial domain, and at least twice spatially differentiable everywhere except at the moving boundary, the reference error, $\tilde{T}(x,t)$, inherits these properties. Therefore, $T,\overset{‾}{T},\tilde{T}$ are continuous and piecewise continuously differentiable, i.e., they are in the Sobolev space $H^{1}(0,\ell )$.

Subtracting the reference system from PDE (1)–(4) yields

(16) $${\tilde{T}}_{t}(x,t)=a{\tilde{T}}_{xx}(x,t),\;x\in (0,\ell )\backslash (s,\bar{s})$$

(17) $$\begin{array}{cc} {\tilde{T}}_{x}(0,t)=\tilde{u}(t), & {\tilde{T}}_{x}(\ell ,t)=0. \end{array}$$

Also, since solutions to (1)–(4) are twice spatially differentiable away from the solidification front, they must have continuous first spatial derivatives. Thus, if $s(t)\neq \overset{‾}{s}(t)$, then ${\overset{‾}{T}}_{x}\left(s^{+}(t),t\right)=$ ${\overset{‾}{T}}_{x}\left(s^{-}(t),t\right)$, and the following relations hold:

(18) $$\dot{s}(t)=b\left({\tilde{T}}_{x}\left(s^{-}(t),t\right)-{\tilde{T}}_{x}\left(s^{+}(t),t\right)\right)$$

(19) $$\overset{\cdot }{\bar{s}}(t)=b({\tilde{T}}_{x}\left({\bar{s}}^{+}(t)-{\tilde{T}}_{x}\left({\bar{s}}^{-}(t)\right)\right).$$

In [25], feedback control law based on temperature that ensures bounded temperature error in the $L_{2}$ norm is derived. Both the temperature and the solidification front appear to be converging to the reference under that control law. However, the control signal is noisy, and not realistically implementable. Therefore, the enthalpy approach is considered.

### C. Enthalpy Formulation and the Choice of the Stefan Problem State

The thermodynamic energy of the material is called enthalpy. In a single-phase material, the enthalpy is approximately proportional to the temperature, with the constant of proportionality equal to $c_{p}$, the specific heat. However, for a solidifying pure material, there is a step change in enthalpy at the melting temperature, $T_{f}$, equal to the latent heat, $L_{f}$. Altogether, this means the enthalpy, denoted by h, can be described as a function of temperature:

(20) $$h(T):=\{\begin{array}{ll} c_{p}T, & T<T_{f}\\ c_{p}T+L_{f}, & T>T_{f}. \end{array}$$

**Fig. 2.**
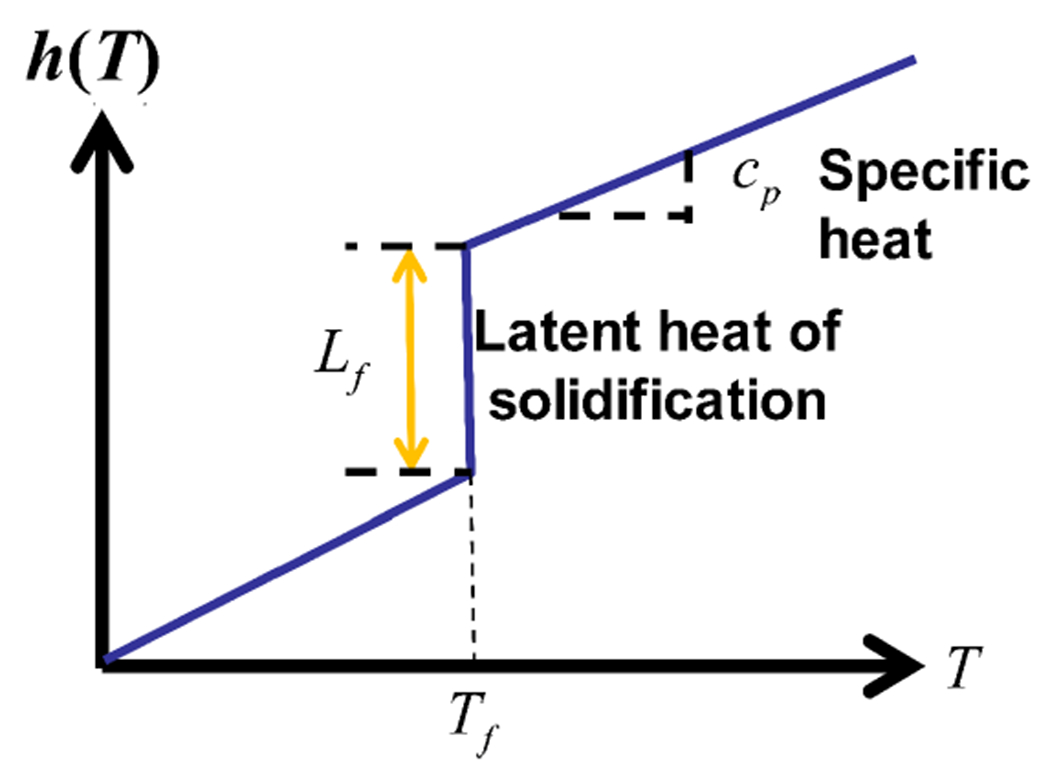
Relationship between enthalpy h and temperature T.

This is illustrated in Fig. 2. In system-theoretic terminology, enthalpy is the actual state variable of the system. In fact, the following PDE is physically equivalent to the Stefan problem:

(21) $${\left(\rho h(T(x,t))\right)}_{t}=kT_{xx}(x,t),\;x\in (0,\ell )$$

(22) $$T_{x}(0,t)=u(t),\;T_{x}(\ell ,t)=0$$

(23) $$T(x,0)=T_{0}(x).$$

The weak forms of the two PDEs (1)–(3) under ODE constraint (4), and (21)–(23) are the same, as discussed in [41]. Although much easier to write down, the enthalpy PDE is more difficult to analyze mathematically due to the discontinuity in the function h(T). In [42], the authors prove that the state operator forms a nonlinear semigroup in $L^{1}$, but this does not necessarily imply that a strong solution to the PDE exists. One advantage of this formulation, though, is that it is easier to simulate numerically. In fact, the simulation code used in this article is based on this PDE, not the true Stefan problem.

Considering the enthalpy error, rather than the temperature, for a Lyapunov functional will result in a much simpler control law that nonetheless performs better than the previous result. However, for clarity of the controller design as it relates to the potentially measurable physical quantities, we take as the full Stefan problem state th e pair (T(x,t),s(t)) of the dependent variables of the PDE/ODE (1)–(4) form, and then relate this pair to enthalpy for the Lyapunov-based stability analysis.

From here on, the notation will be simplified for clarity and space saving, using T(x) to represent T(x,t) or omitting both arguments altogether and using s to represent s(t).

### D. Preliminaries and Notation

If the solution (T,s) to the PDE (1)–(4) satisfies assumptions (A1), (A2), and (A3), a few facts immediately follow. First, $T_{0}(x)<T_{f}$ and is nondecreasing for all 0≤x<s(t) (by weak maximum principle, see [38], Th. 8, p. 389], T(x,t) attains its minimum value on parabolic boundary, i.e., the minimum value is attained on t=0 or x=s(t), which in turn implies $\dot{s}(t)\geq$ 0 for all t≥0. Since (1) is parabolic on the subdomains, if (A3) holds then $T_{x}$ is uniformly bounded (see, e.g., [43], Th. 11.1 from Sec. III.11, p. 211]) by a constant depending on the initial condition and bounds on u. By (4), this means that the solidification front speed is bounded, i.e.,

(24) $$0\leq v_{\text{min}}\leq \dot{s}\leq v_{\text{max}}<\infty .$$

Also, as a consequence of Poincaré’s inequality, there is a bound on $∥T{∥}_{2}$

(25) $$\int_{0}^{\ell }T^{2}dx\leq 2T^{2}(s)+4\ell^{2}\int_{0}^{\ell }T_{x}^{2}dx=2T_{f}^{2}+4\ell^{2}\int_{0}^{\ell }T_{x}^{2}dx.$$

That is, both T and $T_{x}$ are bounded in the $L_{2}(0,\ell )$ norm, and hence, T is bounded in the Sobolev space $H^{1}(0,\ell )$. Similarly, Agmon’s inequality ensures that |T| is also uniformly bounded.

Denote the “enthalpy function” η(T) as

(26) $$\eta (T):=\{\begin{array}{ll} \frac{1}{a}T, & T<T_{f}\\ \frac{1}{a}T+\frac{1}{b}, & T\geq T_{f}. \end{array}$$

The quantity η is proportional to the physical enthalpy h discussed earlier. In this section, we will use the notation $\tilde{\eta }:=$ $\eta (T)-\eta (\tilde{T})$ for the difference in enthalpy, and

(27) $$\tilde{H}:=\int_{0}^{\ell }\tilde{\eta }dx=\int_{0}^{\ell }(\eta (T)-\eta (\bar{T}))dx\;=\frac{1}{a}\int_{0}^{\ell }Tdx+\frac{1}{b}(\ell -s)-\frac{1}{a}\int_{0}^{\ell }\bar{T}dx-\frac{1}{b}(\ell -\bar{s})\;=\frac{1}{a}\int_{0}^{\ell }\tilde{T}dx-\frac{1}{b}\tilde{s}$$

for the total difference. Taking the time derivative of this value, and using the fact that $\tilde{T}$ is continuous

$$\frac{d\tilde{H}}{dt}=\frac{d}{dt}\left(\frac{1}{a}\int_{0}^{\ell }\tilde{T}dx-\frac{1}{b}\tilde{s}\right)=\frac{1}{a}\int_{0}^{\ell }{\tilde{T}}_{t}dx-\frac{1}{b}\overset{\cdot }{\tilde{s}}$$

Denote $s_{1}(t)=min\{s(t),\overset{‾}{s}(t)\}$, and $s_{2}(t)=max\{s(t),\overset{‾}{s}(t)\}$. Using equations (16)–(19) yields

(28) $$\frac{d\tilde{H}}{dt}=\int_{0}^{\ell }{\tilde{T}}_{xx}dx-\frac{1}{b}\dot{s}+\frac{1}{b}\overset{\cdot }{\bar{s}}\;={{\tilde{T}}_{x}|}_{0}^{s_{1}^{-}}+{{\tilde{T}}_{x}|}_{s_{1}^{+}}^{s_{2}^{-}}+{{\tilde{T}}_{x}|}_{s_{2}^{+}}^{\ell }+{{\tilde{T}}_{x}|}_{s^{-}}^{s^{+}}+{{\tilde{T}}_{x}|}_{{\bar{s}}^{-}}^{{\bar{s}}^{+}}\;=-{\tilde{T}}_{x}(0)+{\tilde{T}}_{x}(\ell )=-\tilde{u}.$$

### E. Control Law

With these estimates in mind, we can state the main result of this section. We first present a key technical lemma.

*Lemma II.3 (Precompactness of solutions):* $W^{1,2}(0,\ell )$, the Sobolev space of functions with weak first derivatives bounded in the 2-norm, can be embedded compactly in $C^{0}(0,\ell )$, the space of continuous functions under the supremum norm.

*Proof:* By [38], Th. 5.6 in §5.6.3], we find that any function $u\in W^{1,2}(0,\ell )$ satisfies $u\in C^{0,1/2}(0,\ell )$, where $C^{0,1/2}(0,\ell )$ is the space of Hölder continuous functions with exponent 1/2. This implies that $W^{1,2}(0,\ell )\subset C^{0,1/2}(0,\ell )$.

To show that $C^{0,1/2}(0,\ell )$ is compactly embedded in $C^{0}(0,\ell )$, we wish to apply the Arzelà–Ascoli criterion (see [38], §C.8, p. 718]). This condition requires that the function space on which it is applied is uniformly equicontinuous. We prove this for all Hölder spaces $C^{0,\gamma }(0,\ell )$, for γ∈(0,1].

Given some M≥0, consider $u\in U_{M}$, where

$$U_{M}:=\left\{f\in C^{0,\gamma }(0,\ell ):\left\|f\right\|C^{0,\gamma }\leq M\right\}.$$

From this, we can show that the conditions for uniform equicontinuity are satisfied. Indeed, consider some ϵ>0, as well as any two points x,y∈R, such that |x-y|<δ, where $\delta =:(\epsilon /M{)}^{1/\gamma }$. Any two such points will satisfy

$$\left|u(x)-u(y)\right|\leq {\left|x-y\right|}^{\gamma }M<M_{\epsilon }/M=\epsilon .$$

This proves that the functions in $U_{M}$ are uniformly equicontinuous for any M≥0. Therefore, $C^{0,\gamma }(0,\ell )={⋃}_{M\geq 0}\;U_{M}$ is uniformly equicontinuous, and the Arzelà–Ascoli criterion holds.

It trivially follows from the Arzelà–Ascoli criterion that $C^{0,\gamma }(0,\ell )$ is compactly embedded in $C^{0}(0,\ell )$. Hence, $W^{1,2}(0,\ell )$ is compactly embedded in $C^{0}(0,\ell )$. ∎

*Theorem II.3:* Suppose the initial conditions satisfy assumption (A1) and (A2), the reference temperature profile satisfies assumption (A1)–(A3), the boundary condition satisfies the control law

(29) $$u(t)=\bar{u}(t)+K\tilde{H}(t)=\bar{u}(t)+K\left[\frac{1}{a}\int_{0}^{\ell }\tilde{T}dx-\frac{1}{b}\tilde{s}\right]$$

where K>0, and the closed-loop system satisfies assumption (A3). Then, the reference temperature error $\tilde{T}$ converges asymptotically to 0 uniformly over the domain, and the interface position error $\tilde{s}$ converges to 0 asymptotically as well.

*Proof:* In light of (28), if the control law (29) is used, $\left|\tilde{H}\right|$ and $\left|\tilde{u}\right|$ are exponentially decreasing. As noted earlier, if all assumptions are satisfied, T and $\overset{‾}{T}$, and consequently also $\tilde{T}$ are bounded in $H^{1}(0,\ell )$ over time. Then, by the definition of $\tilde{H}$ in (27), $\left|\tilde{s}\right|$ must also be bounded.

The key to the proof is using an infinite-dimensional invariance principle from [44] to show convergence. Consider the Lyapunov functional candidate

(30) $$V(\tilde{T}):=\frac{1}{2}\int_{0}^{\ell }{\tilde{T}}^{2}dx-\frac{a}{b}T_{f}(s+\bar{s})+\frac{2a}{b}T_{f}\ell$$

on the state space of the error system, $(\tilde{T},\tilde{s})\in H^{1}(0,\ell )\times R$. This function is clearly continuous on the latter space, and non-negative on trajectories of the system. Taking the time derivative of the first term in (30)

(31) $$\frac{d}{dt}\frac{1}{2}\int_{0}^{\ell }{\tilde{T}}^{2}dx=\frac{1}{2}\frac{d}{dt}\left[\int_{0}^{s_{1}}{\tilde{T}}^{2}dx+\int_{s_{1}}^{s_{2}}{\tilde{T}}^{2}dx+\int_{s_{2}}^{\ell }{\tilde{T}}^{2}dx\right]\;=\int_{0}^{s_{1}}\tilde{T}{\tilde{T}}_{t}dx+\int_{s_{1}}^{s_{2}}\tilde{T}{\tilde{T}}_{t}dx+\int_{s_{2}}^{\ell }\tilde{T}{\tilde{T}}_{t}dx.$$

Then, applying PDE (16), and integrating by parts

$$\frac{d}{dt}\frac{1}{2}\int_{0}^{\ell }{\tilde{T}}^{2}dx=a\int_{0}^{s_{1}}\tilde{T}{\tilde{T}}_{xx}dx+a\int_{s_{1}}^{s_{1}}\tilde{T}{\tilde{T}}_{xx}dx+a\int_{s_{2}}^{\ell }\tilde{T}{\tilde{T}}_{xx}dx\;={a\tilde{T}{\tilde{T}}_{x}|}_{0}^{s_{1}^{-}}-a\int_{0}^{s_{1}}{\tilde{T}}_{x}^{2}dx+{a\tilde{T}{\tilde{T}}_{xx}|}_{s_{1}^{+}}^{s_{2}^{-}}-a\int_{s_{1}}^{s_{2}}{\tilde{T}}_{x}^{2}dx+{a\tilde{T}{\tilde{T}}_{x}|}_{s_{2}^{+}}^{\ell }-a\int_{s_{2}}^{\ell }{\tilde{T}}_{x}^{2}dx\;=-a\tilde{T}(0){\tilde{T}}_{x}(0)-{a\tilde{T}{\tilde{T}}_{xx}|}_{{\bar{s}}^{-}}^{{\text{bars}}^{+}}-{a\tilde{T}{\tilde{T}}_{xx}|}_{s^{-}}^{s^{+}}a\tilde{T}\left(\ell \right){\tilde{T}}_{x}(\ell )-a\int_{0}^{\ell }{\tilde{T}}_{x}^{2}dx.$$

Applying (17)–(19)

(32) $$\frac{d}{dt}\frac{1}{2}\int_{0}^{\ell }{\tilde{T}}^{2}dx=-a\tilde{T}(0)\tilde{u}-a\int_{0}^{\ell }{\tilde{T}}_{x}^{2}dx+\frac{a}{b}\tilde{T}(s)\dot{s}-\frac{a}{b}\tilde{T}(\bar{s})\overset{\cdot }{\bar{s}}.$$

Differentiating the second term in (30) gives

(33) $$\frac{d}{dt}\frac{a}{b}T_{f}(s+\bar{s})=\frac{a}{b}T_{f}(\dot{s}+\overset{\cdot }{\bar{s}}).$$

Combining (32) and (33)

(34) $$\frac{d}{dt}V(\tilde{T})=-a\tilde{T}(0)\tilde{u}-a\int_{0}^{\ell }{\tilde{T}}_{x}^{2}dx+\frac{a}{b}\left(\tilde{T}(s)-T_{f}\right)\dot{s}-\frac{a}{b}\left(\tilde{T}(\bar{s})+T_{f}\right)\overset{\cdot }{\bar{s}}\;=-a\tilde{T}(0)\tilde{u}-a\int_{0}^{\ell }{\tilde{T}}_{x}^{2}dx-\frac{a}{b}(\bar{T}(s)\dot{s}+T(\bar{s})\overset{\cdot }{\bar{s}}).$$

It was already noted that $\left|\tilde{u}\right|$ decreases exponentially, and

(35) $$|\tilde{T}(0)|=|\tilde{T}(0)-\tilde{T}(\ell )|=\left|\int_{0}^{\ell }{\tilde{T}}_{x}dx\right|\leq \sqrt{\ell }{\left\|{\tilde{T}}_{x}\right\|}_{2}$$

where the final inequality follows from the Cauchy–Schwarz inequality, given that the first term is exponentially decreasing. From the discussion in Section II, it follows that $\tilde{T}(\ell )=0$ for all times, since $T(\ell )=\overset{‾}{T}(\ell )=T_{f}$. Under the assumptions, both $\dot{s}$ and $\dot{\overset{‾}{s}}$ are positive and bounded below, as discussed earlier. Since the temperatures are bounded, choosing an appropriate temperature scale ensures $\overset{‾}{T}(s)$ and $T(\overset{‾}{s})$ are also nonnegative. So, after a sufficiently long time interval

(36) $$\frac{d}{dt}\tilde{V}(t)\leq -a\int_{0}^{\ell }{\tilde{T}}_{x}^{2}dx.$$

Then, applying Poincaré’s inequality

(37) $$-a\int_{0}^{\ell }{\tilde{T}}_{x}^{2}dx\leq -\frac{a}{4\ell^{2}}\int_{0}^{\ell }{\tilde{T}}^{2}dx+2{\tilde{T}}^{2}(\ell )\;=-\frac{a}{4\ell^{2}}\int_{0}^{\ell }{\tilde{T}}^{2}dx=:-W(\tilde{T}).$$

Now we will verify the technical conditions required for the application of the invariance principle ([44], Th. 6.3, p.195]). Using the notation of [44], let $𝒳=H^{1}(0,\ell )\times R$ and 𝒴= $C^{0}(0,\ell )\times R$, and let f(x) be an admissible initial value for the reference error. That is, $f(x)=T_{0}-{\overset{‾}{T}}_{0}$, where $T_{0},{\overset{‾}{T}}_{0}$ satisfy assumptions (A1) and (A2).

Define $𝒢:=\gamma (f):={⋃}_{t\geq 0}\;\{S(t)f\}$, where S(t)f is the solution to the error equations (16)–(17) under the given control law (29). Since solutions to the Stefan problem are continuous and piecewise $C^{2},𝒢\subset 𝒴$. According to Lemma 2.3, 𝒳 can be compactly embedded in 𝒴. Therefore, 𝒢 is compactly embedded in 𝒴 and, as noted earlier, 𝒢 is 𝒳-bounded. Define $\overset{ˆ}{W}$ and $\overset{ˆ}{V}$ to be the extensions of W and V to ${Cl}_{𝒴}(𝒢)$, i.e., the closure of 𝒢 in the supremum norm. Since functions in 𝒢 will be twice differentiable almost everywhere, both of these functionals are well defined, positive semidefinite, and lower semicontinuous on ${Cl}_{𝒴}(𝒢)$. Thus, all conditions of [44], Th. 6.3, p. 195] are met, which yields the following result:

(38) $$\underset{t\to \infty }{\lim}d_{𝒴}\left(S(t)f,{ℳ}_{3}\right)=0$$

where ${ℳ}_{3}\subset \left\{y\in {Cl}_{𝒴}(𝒢):\overset{ˆ}{W}(y)=0\right\}$.

In general, ${ℳ}_{3}:=\left\{\tilde{T}(x):{\tilde{T}}_{x}(x)=0\right\}$, that is T(x)= $\overset{‾}{T}(x)+C$ for some constant C. So, consider any constant element $\tilde{T}(x):=C$ in 𝒢. If C≠0, then $s\neq \overset{‾}{s}$, but since T is continuously differentiable except at $s,{\overset{‾}{T}}_{x}\left({\overset{‾}{s}}^{+}\right)=T_{x}\left({\overset{‾}{s}}^{+}\right)=$ $T_{x}\left({\overset{‾}{s}}^{-}\right)={\overset{‾}{T}}_{x}\left({\overset{‾}{s}}^{-}\right)$. Then, by (4), $\dot{\overset{‾}{s}}=-b\left({\overset{‾}{T}}_{x}\left({\overset{‾}{s}}^{+}\right)-{\overset{‾}{T}}_{x}\left({\overset{‾}{s}}^{+}\right)\right)$. This contradicts assumption (A4). This means that ${ℳ}_{3}\cap 𝒢=$ {0}, and since ${ℳ}_{3}\subset {Cl}_{𝒴}(𝒢),{ℳ}_{3}=\{0\}$. Therefore, (38) is equivalent to ${lim}_{t\to \infty }\;∥\tilde{T}(x,t){∥}_{\infty }=0$.

Finally, since both $\tilde{T}$ and $\left|\tilde{H}\right|$ converge to 0, according to the definition (27), $\tilde{s}$ must converge to 0 as well. ∎

**Fig. 3.**
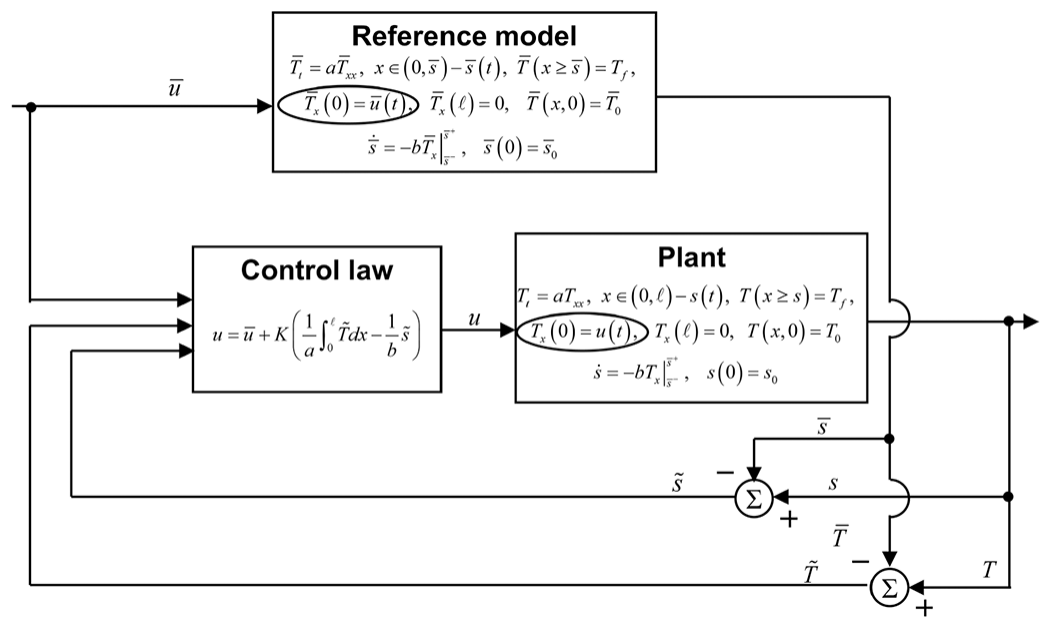
Block diagram for the control law with the full-state enthalpy feedback and its calculations in terms of temperature and solidification front position.

This control algorithm is summarized in the block diagram form in Fig. 3. The control law uses enthalpy which cannot be directly measured, but which is calculated as given in (29). The resulting control signal then becomes smooth and implementable, as will be demonstrated in numerical simulations below, unlike that in the temperature-based controller proposed in [25].

### F. Simulation Results and Discussion

Assumptions (A1) and (A2) depend only on the initial conditions and are entirely reasonable. Assumption (A3) can be satisfied by choosing the controller gain K sufficiently small. For example

(39) $$K<\frac{\inf_{t}\bar{u}(t)}{{\tilde{H}|}_{t=0}}$$

will ensure that assumption (A3) holds for u. This gain can be further adjusted to limit the amount of control actuation as the tradeoff in reducing the convergence rate. The development abovementioned assumes that 0<s(t)<ℓ. In the cases when s(t)=0 or s(t)=ℓ the system is linear, and the control law can still be used.

**Fig. 4.**
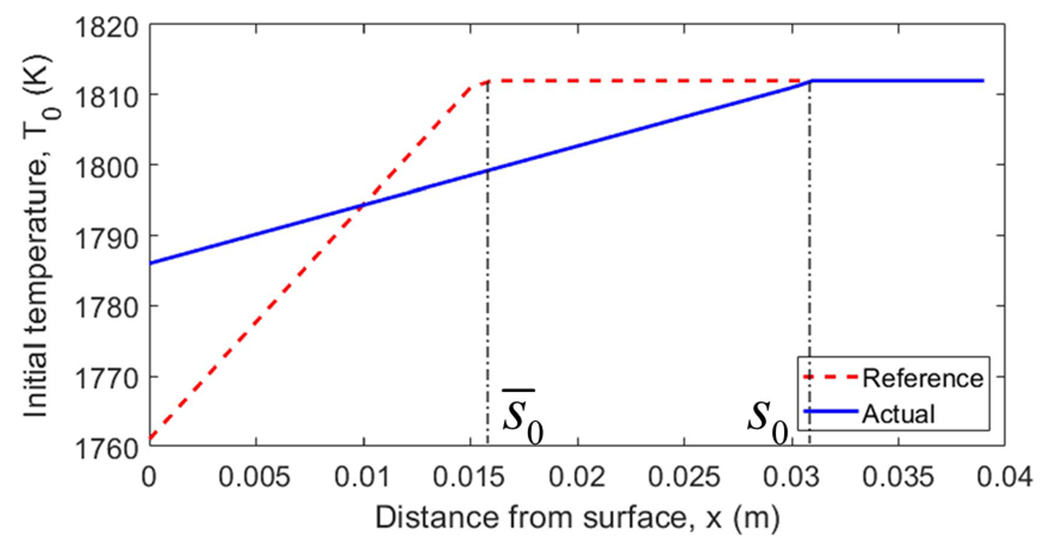
Initial condition for simulations.

**Fig. 5.**
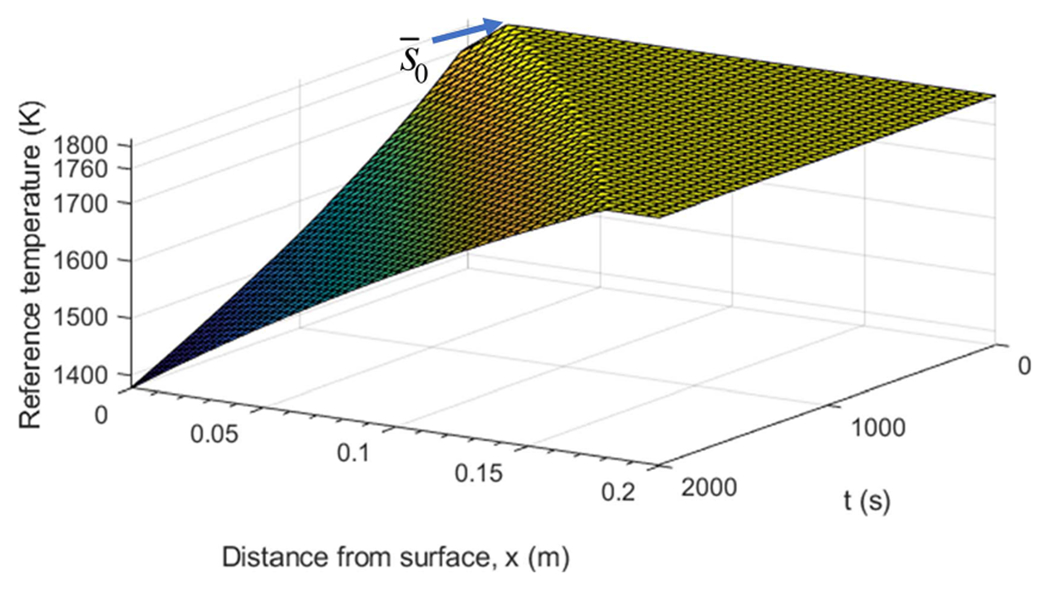
Reference temperature $\overset{‾}{T}(x,t)$ and solidification front position $\overset{‾}{s}(t)$ used in the trajectory tracking simulations.

**Fig. 6.**
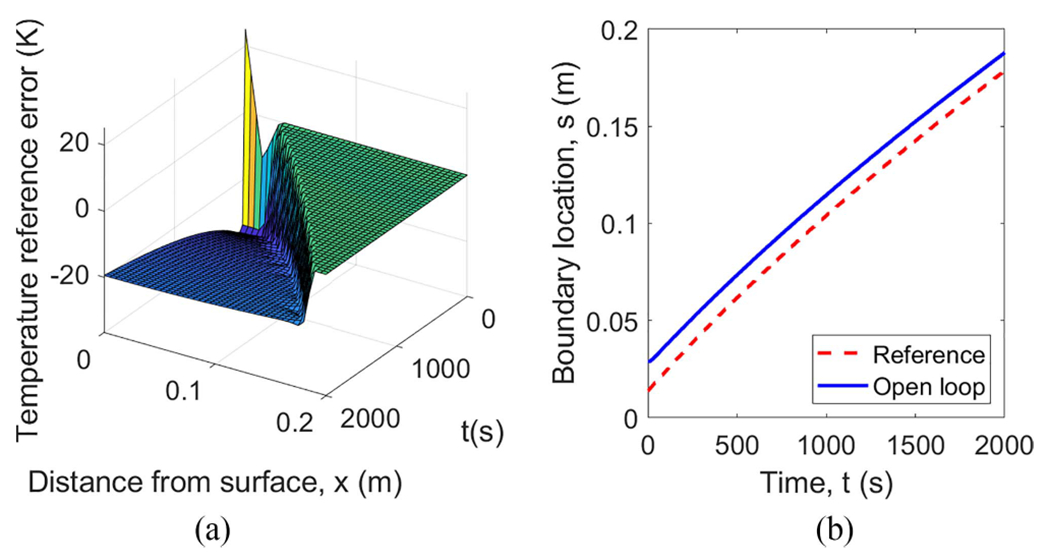
Simulation of the open-loop system (1)–(4) with initial condition mismatch shown in Fig. 4 and $u(t)=\overset{‾}{u}(t)$. (a) Reference temperature error $\overset{‾}{T}(x,t)$. (b) Solidification front s(t).

**Fig. 7.**
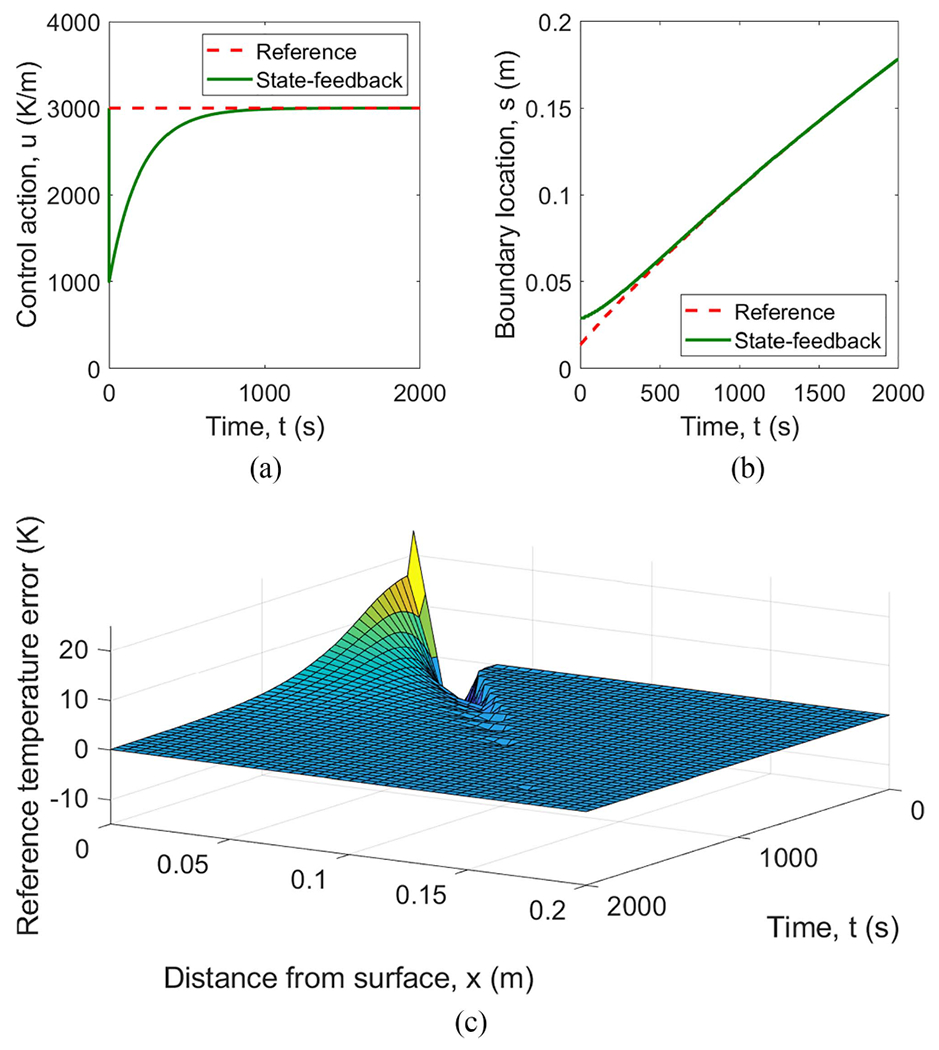
Simulation of system (1)–(4) with initial condition mismatch in Fig. 4 under feedback control law (29). (a) Boundary control u(t) from (29). (b) Solidification front s(t). (c) Reference temperature error T(x, t).

The specific initial conditions used in the simulation are shown in Fig. 4. The reference temperature $\overset{‾}{T}(x,t)$ and the solidification front $\overset{‾}{s}(t)$ are shown in Fig. 5, where the top flat surface depicts the liquid phase corresponding to the melting temperature $T_{f}$, the crease to the left of the flat top shows the solidification front position along x-axis as the function of time, and the surface starting right below the flat top and going downward corresponds to the spatially distributed solid phase temperature. To better cross-reference Figs. 4 and 5, solidification front position ${\overset{‾}{s}}_{0}$ at t=0 is marked in both. The other simulation parameters are given in Table I. Fig. 6 shows the behavior of the system under open-loop control with $u(t)=\overset{‾}{u}(t)$ for all t≥0. In this case, the reference errors in both temperature and solidification front position appear to converge to constant, non-zero values. Fig. 7 shows the performance of the system under the control law (29). Clearly, the temperature profile converges to the desired reference. Furthermore, the temperature and the solidification front appear to be converging exponentially fast, but this is not guaranteed by the proof.

## III. Estimation and Output Feedback

In the casting process, only the surface temperature could realistically be measured. So, the problem to resolve in this section is to find a control law that works with boundary (Dirichlet) sensing.

### A. Observer Design

Let $\overset{ˆ}{T}$ and $\overset{ˆ}{s}$ be estimates of the temperature T and the interface location s, respectively, satisfying the following PDE:

(40) $${\hat{T}}_{t}(x,t)=a{\hat{T}}_{xx}(x,t),\;x\in (0,\ell )\backslash \{\hat{s}(t)\}$$

(41) $$\hat{T}(\hat{s}(t),t)=T_{f},\;{\hat{T}}_{x}(0,t)=u(t),\;{\hat{T}}_{x}(\ell ,t)=0$$

(42) $$\hat{T}(x,0)={\hat{T}}_{0}(x),\;\hat{s}(0)={\hat{s}}_{0}$$

(43) $$\overset{\cdot }{\hat{s}}(t)=-b\left({\hat{T}}_{x}\left({\hat{s}}^{+}(t)\right)-{\hat{T}}_{x}\left({\hat{s}}^{-}(t)\right)\right)+l(\hat{T}(0,t)-T(0,t))$$

where the initial conditions satisfy assumption (A1), and the estimation gain l is strictly positive. Intuitively, the estimation scheme (43) can be thought of as lowering the latent heat, or equivalently lowering the enthalpy in the liquid subdomain.

*Theorem III.1:* Consider the observer system (40)–(43). Suppose the initial conditions satisfy assumptions (A1) and (A2), and the boundary flux satisfies assumption (A3). Then, with a sufficiently small estimation gain $l,\overset{ˆ}{T}$ converges asymptotically to T uniformly over the domain, and $\overset{ˆ}{s}$ converges asymptotically and monotonically to s.

*Proof:* In light of the proof of Theorem 2.3, consider the following Lyapunov function candidate:

(44) $$V(\hat{T}-T):=\frac{1}{2}\int_{0}^{\ell }{\left(\hat{T}-T\right)}^{2}dx-\frac{a}{b}T_{f}(\hat{s}+s)+\frac{2a}{b}T_{f}\ell .$$

Taking the time derivative of (44) yields

(45) $$\dot{V}=-a\int_{0}^{\ell }{\left({\hat{T}}_{x}-T_{x}\right)}^{2}dx-\frac{a}{b}(T(\hat{s})\overset{\cdot }{\hat{s}}+\hat{T}(s)\dot{s})\;-\frac{l}{b}\left(\hat{T}(\hat{s})-T(\hat{s})\right)\left(\hat{T}(0)-T(0)\right).$$

By the weak maximum principle, ${\overset{ˆ}{T}}_{x}\left({\overset{ˆ}{s}}^{-}\right)\geq 0$. Then, choosing

(46) $$l\leq \frac{b\underset{t}{\text{min}}\left|{\hat{T}}_{x}\left({\hat{s}}^{-}(t)\right)\right|}{\underset{t}{\text{max}}|\hat{T}(0,t)-T(0,t)|}$$

ensures that $\dot{\overset{ˆ}{s}}\geq 0$. Choosing an appropriate temperature scale to ensure that $\overset{ˆ}{T}(s)$ and $T(\overset{ˆ}{s})$ are nonnegative yields

(47) $$\dot{V}\leq -a\overset{\ell }{\underset{0}{\int }}{\left({\hat{T}}_{x}-T_{x}\right)}^{2}dx-\frac{l}{b}\left(T_{f}-T(\hat{s})\right)(\hat{T}(0)-T(0)).$$

Then, applying Poincaré’s inequality gives

(48) $$\dot{V}\leq -\frac{a}{4\ell^{2}}\int_{0}^{\ell }{\left(\hat{T}-T\right)}^{2}dx-\frac{l}{b}\left(T_{f}-T(\hat{s})\right)(\hat{T}(0)-T(0)).$$

Since $T(\overset{ˆ}{s})\leq T_{f}$, if $(\overset{ˆ}{T}-T)(0)\geq 0$

(49) $$\dot{V}\;\leq -\frac{a}{4\ell^{2}}\int_{0}^{\ell }{\left(\hat{T}-T\right)}^{2}dx\;\leq -\frac{a}{8\ell^{2}}\int_{0}^{\ell }{\left(\hat{T}-T\right)}^{2}dx=:-W(\hat{T}-T).$$

If $(\overset{ˆ}{T}-T)(0)<0$, similarly to (35)

(50) $$|(\hat{T}-T)(0)|\leq \sqrt{\ell }{\left\|{\hat{T}}_{x}-T_{x}\right\|}_{2}.$$

Therefore,

(51) $$\dot{V}\leq -\frac{a}{4\ell^{2}}\int_{0}^{\ell }{\left(\hat{T}-T\right)}^{2}dx+\frac{l\sqrt{\ell }C_{1}}{b}{\left\|{\hat{T}}_{x}-T_{x}\right\|}_{2}$$

where $C_{1}={max}_{t}\;\left(T_{f}-T(\overset{ˆ}{s}(t))\right)>0$.

As mentioned in Section II-D, $T_{x}$ is uniformly bounded by a constant depending on the initial condition and bounds on u. Therefore, there exists ad constant $C_{2}$ such that

(52) $${\left\|{\hat{T}}_{x}-T_{x}\right\|}_{2}\leq C_{2}.$$

From this fact, we find

(53) $$\dot{V}\leq -\frac{a}{4\ell^{2}}\int_{0}^{\ell }{\left(\hat{T}-T\right)}^{2}dx+\frac{l\sqrt{\ell }C_{1}C_{2}}{b}.$$

Obviously, $\left|\overset{ˆ}{T}-T\right|$ is bounded below by 0, and there exists an infimum. Choose

(54) $$l\leq \frac{ab}{8\ell^{2.5}C_{1}C_{2}}\text{inf}|\hat{T}-T{|}^{2}.$$

Then, (53) becomes

(55) $$\dot{V}\leq -\frac{a}{4\ell^{2}}\int_{0}^{\ell }{\left(\hat{T}-T\right)}^{2}dx+\frac{ab}{8\ell^{2.5}C_{1}C_{2}}\text{inf}|\hat{T}-T{|}^{2}\frac{\sqrt{\ell }C_{1}C_{2}}{b}\;\leq -\frac{a}{4\ell^{2}}\int_{0}^{\ell }{\left(\hat{T}-T\right)}^{2}dx+\frac{a}{8\ell^{2}}\text{inf}|\hat{T}-T{|}^{2}\;\leq -\frac{a}{8\ell^{2}}\int_{0}^{\ell }{\left(\hat{T}-T\right)}^{2}dx=:-W(\hat{T}-T).$$

Combining (46) and (54), and choosing a sufficiently small l that satisfies both inequalities while ensuring that $\dot{V}(t)$ satisfies (55) for all t. Applying the invariance principle as in the proof of Theorem 2.3, verifying that Lemma 2.3 holds, guarantees that $\overset{ˆ}{T}-T$ converges to 0 uniformly.

To prove convergence of the solidification front estimate, assume that we have attained uniform convergence of the temperature field, i.e., $∥T-\overset{ˆ}{T}{∥}_{\infty }=0$, while at the same time we have $|s-\overset{ˆ}{s}|>0$. We identify the following two cases:

1. $\overset{ˆ}{s}<s$, i.e., there is assumed to be a liquid region in the solid domain;
2. $\overset{ˆ}{s}>s$, i.e., there is assumed to be a solid region in the liquid domain.

Our goal is to prove that both cases are unstable, driving $\overset{ˆ}{s}\to s$ by virtue of the sign of $\dot{s}$ and the properties of T.

Consider first case i). We clearly find that there exists some ϵ>0 and

$$\exists x_{1}<\hat{s},\;{\hat{T}}_{x}\left(x_{1}\right)>\epsilon >0\;\exists x_{2}\geq s,\;{\hat{T}}_{x}\left(x_{2}\right)=0.$$

Then, application of the weak maximum principle yields ${\overset{ˆ}{T}}_{x}\left(s^{-}\right)>{\overset{ˆ}{T}}_{x}\left(s^{+}\right)>0.\;$ Recalling that $\dot{s}=-b\left[{\overset{ˆ}{T}}_{x}\left({\overset{ˆ}{s}}^{+}\right)-\right.\;\left.{\overset{ˆ}{T}}_{x}\left({\overset{ˆ}{s}}^{-}\right)\right]+l[\overset{ˆ}{T}(0)-T(0)]$, the latter inequality implies that $\dot{\overset{ˆ}{s}}>0$, i.e., the solidification front is driven toward s. In the dynamics of the observer’s solidification front, $|\overset{ˆ}{T}(0)-T(0)|=0$ given that we assume $∥T-\overset{ˆ}{T}{∥}_{\infty }=0$.

**Fig. 8.**
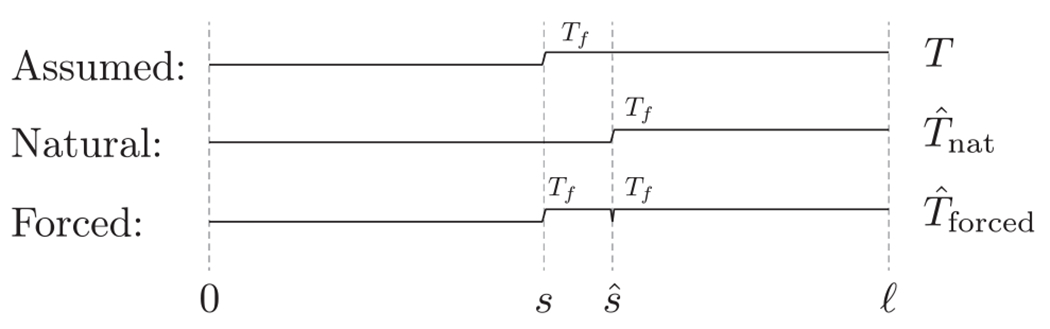
Illustration of the contradiction for case ii) in Theorem 3.1. Since it is assumed that $∥T-\overset{ˆ}{T}{∥}_{\infty }=0,\overset{ˆ}{T}$ cannot assume the form of ${\overset{ˆ}{T}}_{\text{nat}}$, and is forced to assume the shape of ${\overset{ˆ}{T}}_{\text{forced}}$, which implies the existence of a phase change interface at s and $\overset{ˆ}{s}$. This contradicts the uniform convergence argument, and if it were to exist, it would imply $\dot{\overset{ˆ}{s}}<0$.

Consider now case ii). Since it is assumed that $∥T-\overset{ˆ}{T}{∥}_{\infty }=$ 0, for there to be a phase-change interface at s, it is necessary that $\overset{ˆ}{T}\left({\overset{ˆ}{s}}^{-}\right)<T_{f}$ and $\overset{ˆ}{T}\left({\overset{ˆ}{s}}^{+}\right)\geq T_{f}$. However, since T will have $T(x)\geq T_{f}$ for $x\in (s,\overset{ˆ}{s})$, this implies that we must have ${\overset{ˆ}{T}}_{x}\left({\overset{ˆ}{s}}^{-}\right)<0,{\overset{ˆ}{T}}_{x}\left({\overset{ˆ}{s}}^{+}\right)>0$ to guarantee that there exists a phase-change interface at $\overset{ˆ}{s}$, despite uniform convergence of $\overset{ˆ}{T}$ to T. If this could be the case, we would have ${\overset{ˆ}{T}}_{x}\left({\overset{ˆ}{s}}^{+}\right)>{\overset{ˆ}{T}}_{x}\left({\overset{ˆ}{s}}^{-}\right)$, which implies $\dot{\overset{ˆ}{s}}<0$ (see Fig. 8 for an illustration).

Since the latter temperature field $\overset{ˆ}{T}$ violates the monotonicity assumption of (A3), we find that case ii) cannot be attained in the limit t→∞ under uniform convergence of $\overset{ˆ}{T}$ to T, leading to a contradiction.

Hence, $\overset{ˆ}{s}$ will only converge to a *stable* equilibrium, which for temperature profile T only exists at ${lim}_{t\to \infty }\;s(t)$. By uniform convergence of the temperature profile tracking error profile, we have therefore shown that $\overset{ˆ}{s}\to s$ asymptotically and monotonically. ∎

### B. Output Feedback Controller Design

*Theorem III.2:* Suppose $\left(T,s),(\overset{‾}{T},\overset{‾}{s}\right)$, and $\left(\overset{ˆ}{T},\overset{ˆ}{s}\right)$ all satisfy the hypotheses of Theorem 3.1. Then, under the control law

$$u=\bar{u}+K\left(\frac{1}{a}\int_{0}^{\ell }(\hat{T}-\bar{T})dx-\frac{1}{b}(\hat{s}-\bar{s})\right)$$

the temperature reference error $\tilde{T}$ and the estimation error $\overset{ˆ}{T}-T$ uniformly converge to 0, and s converges to $\overset{‾}{s}$ asymptotically and monotonically.

*Proof:* Denote, respectively, by $\overset{ˆ}{H}$ and $\overset{‾}{H}$, the total estimated and reference enthalpies:

$$\hat{H}=\int_{0}^{\ell }\hat{\eta }dx,\;\bar{H}=\int_{0}^{\ell }\bar{\eta }dx$$

where $\overset{ˆ}{\eta }=\eta (\overset{ˆ}{T})$ and $\overset{‾}{\eta }=\eta (\overset{‾}{T})$, as defined in (26).

The control law can be rewritten as

(57) $$\tilde{u}(t)=K(\hat{H}-\bar{H}).$$

Taking the time derivative yields

(58) $$\overset{\cdot }{\tilde{u}}=K\left[\int_{0}^{\ell }{\left(\hat{T}-\bar{T}\right)}_{xx}-\frac{1}{b}\overset{\cdot }{\hat{s}}+\frac{1}{b}\overset{\cdot }{\hat{s}}\right]\;=K\left[-{\left(\hat{T}-\bar{T}\right)}_{x}(0)+{\left(\hat{T}-\bar{T}\right)}_{x}(\ell )-\frac{l}{b}(\hat{T}(0)-T(0))\right]\;=-K\tilde{u}-\frac{Kl}{b}(\hat{T}(0)-T(0))$$

which implies that $\tilde{u}$ converges to 0. The remainder of the proof is a Lyapunov argument on the moving boundary system, similar to the proof of Theorem 3.1.

Consider the following Lyapunov functional candidate:

(59) $$V(\tilde{T},t):=\frac{1}{2}\int_{0}^{\ell }{\tilde{T}}^{2}dx+\frac{1}{2}\int_{0}^{\ell }{\left(\hat{T}-T\right)}^{2}dx\;-\frac{a}{b}T_{f}\left(\hat{s}+2^{∼}s+\bar{s}\right)+4\frac{a}{b}T_{f}\ell .$$

Taking the time derivative yields

$$\dot{V}=-a\tilde{T}(0)\tilde{u}-a\int_{0}^{\ell }{\tilde{T}}_{x}^{2}dx\;-\frac{a}{b}(\bar{T}(s)\dot{s}+T(\bar{s})\overset{\cdot }{\bar{s}}+T(\hat{s})\overset{\cdot }{\hat{s}}+\hat{T}(s)\dot{s})\;-a\int_{0}^{\ell }{\left(\hat{T}-T\right)}_{x}^{2}dx-\frac{l}{b}\left(T_{f}-T(\hat{s})\right)(\hat{T}-T)(0)\;\leq \frac{a}{4\ell^{2}}\left(\int_{0}^{\ell }{\tilde{T}}^{2}dx+\int_{0}^{\ell }{\left(\hat{T}-T\right)}^{2}dx\right)\;-\frac{l}{b}\left(T_{f}-T(\hat{s})\right)(\hat{T}-T)(0).$$

Following a similar line of reasoning as in the proof of Theorem 3.1, we find

(61) $$\dot{V}\leq -\frac{a}{4\ell^{2}}\left(\int_{0}^{\ell }{\tilde{T}}^{2}dx+\int_{0}^{\ell }{\left(\hat{T}-T\right)}^{2}dx\right).$$

Applying the invariance principle proves uniform convergence of the temperature tracking and estimation errors.

The solidification front convergence proof follows the exact same reasoning as the proof of Theorem 3.1, with the exception that (T,s) should be replaced by $\left(\overset{‾}{T},\overset{‾}{s}\right)$, and $\left(\overset{ˆ}{T},\overset{ˆ}{s}\right)$ should be read as (T,s). From this, it follows that s converges to $\overset{‾}{s}$ asymptotically and monotonically. ∎

**Fig. 9.**
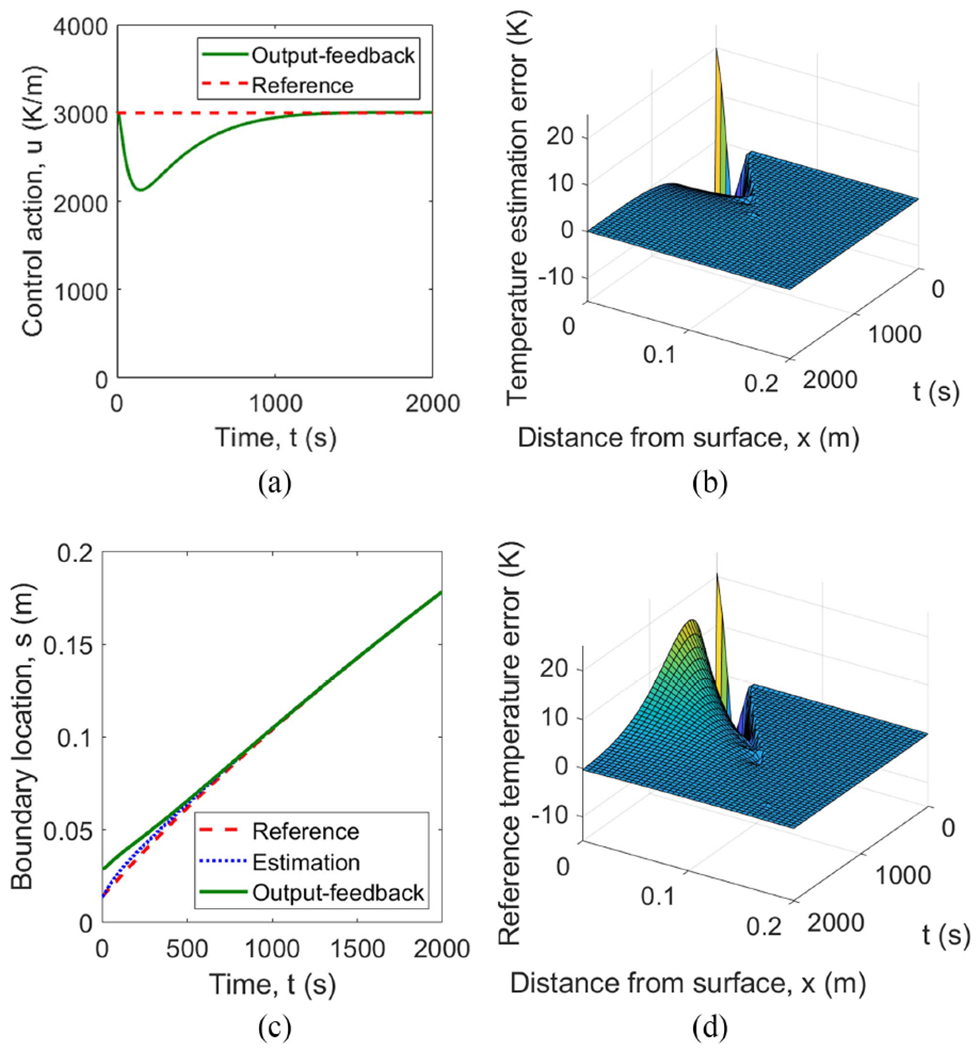
Simulation of system (1)–(4) with initial condition mismatch and output feedback control law (56) with estimator (40)–(43). (a) Boundary control u(t) from (56). (b) Temperature estimation error $T-\overset{‾}{T}(x,t)$. (c) Solidification front s(t). (d) Temperature reference error $\overset{‾}{T}(x,t)$.

Fig. 9 shows a simulation using this output-feedback control law. The initial estimate equals the reference initial conditions, i.e., ${\overset{ˆ}{T}}_{0}={\overset{‾}{T}}_{0}$ and ${\overset{ˆ}{s}}_{0}={\overset{‾}{s}}_{0}$. The errors of the estimates and the actual temperature and solidification front clearly converge to 0, and even appear to be converging exponentially fast, and the control action is seen to remain bounded.

Fig. 13 shows a block diagram of this output-feedback algorithm forming each side of the two-sided control topology. To improve estimation accuracy when boundary sensing is available only at several points along a caster, the authors proposed in [26] an online estimator recalibration method, which uses sparse discrete-in-time temperature measurements.

## IV. Two-Sided Stefan Problem

### A. Full-State Feedback Control

**Fig. 10.**
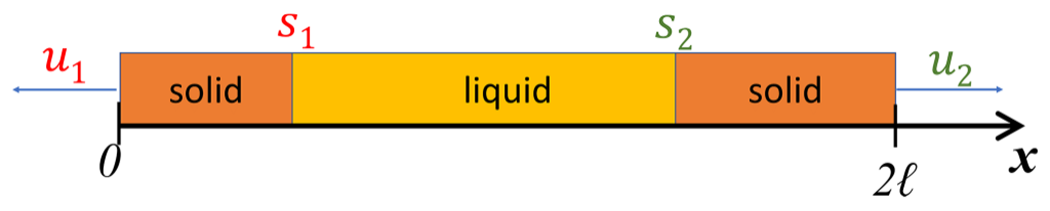
Schematic of the two-sided Stefan problem.

The boundary condition at x=ℓ in (2) assumes that the temperature profile is symmetric about the centerline of a material with length 2ℓ. However, the actual system lacks this symmetry. Therefore, model (1)–(4) should include both sides of the caster, giving two moving solid–liquid boundaries and two Neumann boundary conditions, as shown in Fig. 10:

(62) $$T_{t}(x,t)=aT_{xx}(x,t),\;x\in (0,2\ell )\backslash \left\{s_{1}(t),s_{2}(t)\right\}$$

(63) $$T\left(s_{1}(t),t\right)=T_{f}=T\left(s_{2}(t),t\right)\;T_{x}(0,t)=u_{1}(t),\;T_{x}(2\ell ,t)=-u_{2}(t)$$

(64) $$T(x,0)=T_{0}(x),\;x\in (0,2\ell )$$

(65) $${\dot{s}}_{1}(t)=b\left(T_{x}\left(s_{1}^{-},t\right)-T_{x}\left(s_{1}^{+},t\right)\right),\;s_{1}(0)=s_{1,0}\;{\dot{s}}_{2}(t)=b\left(T_{x}\left(s_{2}^{+},t\right)-T_{x}\left(s_{2}^{-},t\right)\right),\;s_{2}(0)=s_{2,0}$$

Here, the material is liquid between $s_{1}$ and $s_{2}$, and solid otherwise. Assumption (A1) can be adjusted to reflect this.

(A5) The initial conditions satisfy: $0<s_{1,0}<s_{2,0}<2\ell ;\;T_{0}$ is piecewise smooth, continuous, nondecreasing for $x\in \;\left(0,s_{1,0}\right)$, and nonincreasing for $x\in \left(s_{2,0},2\ell \right);T_{0}(x)<T_{f}$ for all $x\in \left(0,s_{1,0}\right)\cup \left(s_{2,0},2\ell \right)$ and $T_{0}(x)=T_{f}$ for all $x\in \;\left(s_{1,0},s_{2,0}\right)$.

As a direct extension of Theorems 2.1 and 2.2, it can be proven that there exists a unique solution to problem (62)–(65) under assumption (A5).

As a direct result of (A5), we have $\dot{s_{1}}\geq 0,\dot{s_{2}}\leq 0$. The reference profiles $\left(\overset{‾}{T}(x,t),\overset{‾}{s}(t)\right)$ and errors $\left(\tilde{T}(x,t),\tilde{s}(t)\right)$ can be defined equivalently

(66) $${\tilde{T}}_{t}(x,t)=a{\tilde{T}}_{xx}(x,t),\;x\in (0,2\ell )\backslash \left\{s_{1}(t),s_{2}(t)\right\}$$

(67) $$\tilde{T}\left({\tilde{s}}_{1}(t),t\right)=T_{f}=\tilde{T}\left({\tilde{s}}_{2}(t)\right)$$

(68) $${\tilde{T}}_{x}(0,t)={\tilde{u}}_{1}(t),\;{\tilde{T}}_{x}(2\ell ,t)=-{\tilde{u}}_{2}(t)$$

(69) $$\begin{array}{cc} \tilde{T}(x,0)={\tilde{T}}_{0}(x), & \;\;x\in (0,2\ell ). \end{array}$$

Similarly to (7) and (19), the following equations can be obtained:

(70) $${\dot{s}}_{1}(t)=-{b{\tilde{T}}_{x}(x,t)|}_{s_{1}^{-}(t)}^{s_{1}^{+}(t)}$$

(71) $${\overset{\cdot }{\bar{s}}}_{1}(t)=-{b{\tilde{T}}_{x}(x,t)|}_{{\bar{s}}_{1}^{-}(t)}^{{\bar{s}}_{1}^{+}(t)}$$

(72) $${\dot{s}}_{2}(t)=-{b{\tilde{T}}_{x}(x,t)|}_{s_{2}^{-}(t)}^{s_{2}^{+}(t)}$$

(73) $${\overset{\cdot }{\bar{s}}}_{2}(t)=-{b{\tilde{T}}_{x}(x,t)|}_{{\bar{s}}_{2}^{-}(t)}^{{\bar{s}}_{2}^{+}(t)}$$

Now, we can extend Theorem 3.1 as follows.

*Theorem IV.1:* Denote

(74) $${\tilde{H}}_{2}:=\int_{0}^{2\ell }\tilde{\eta }dx=\frac{1}{a}\int_{0}^{2\ell }\tilde{T}dx+\frac{1}{b}\left({\tilde{s}}_{2}-{\tilde{s}}_{1}\right).$$

Let the reference and the actual systems satisfy assumptions (A3) and (A5), and the Neumann boundary conditions satisfy

(75) $${\tilde{u}}_{1}={\bar{u}}_{1}+K\left(\frac{1}{a}\int_{0}^{\ell }\tilde{T}dx-\frac{1}{b}{\tilde{s}}_{1}\right)\;{\tilde{u}}_{2}={\bar{u}}_{2}+K\left(\frac{1}{a}\int_{\ell }^{2\ell }\tilde{T}dx+\frac{1}{b}{\tilde{s}}_{2}\right)$$

where gain k>0, so that the control law is given by (75).

Then, the reference temperature error $\tilde{T}$ converges asymptotically to 0 uniformly over the domain, and both interface position errors converge to 0 asymptotically as well.

*Proof:* The proof follows the same basic principle as the proof of Theorem 3.1. Denote ${\tilde{u}}_{1}=u_{1}-{\overset{‾}{u}}_{1},{\tilde{u}}_{2}=u_{2}-{\overset{‾}{u}}_{2}$, we have

(76) $${\tilde{u}}_{1}(t)+{\tilde{u}}_{2}(t)=K{\tilde{H}}_{2}(t).$$

The Neumann boundary control can be shown to directly affect the total enthalpy error

(77) $$\frac{d{\tilde{H}}_{2}}{dt}=-{\tilde{u}}_{1}(t)-{\tilde{u}}_{2}(t)=-K{\tilde{H}}_{2}(t).$$

Therefore, the control law (75) drives the enthalpy error to 0.

The temperature convergence can be shown using the following Lyapunov functional:

(78) $$V(\tilde{T}):=\frac{1}{2}\int_{0}^{2\ell }{\tilde{T}}^{2}dx-\frac{a}{b}T_{f}\left(s_{1}+{\bar{s}}_{1}\right)+\frac{a}{b}T_{f}\left(s_{2}+{\bar{s}}_{2}\right).$$

Taking the time derivative, and using the PDE (62)–(65), gives

(79) $$\dot{V}(\tilde{T})=-a\tilde{T}(0){\tilde{u}}_{1}-a\tilde{T}(2\ell ){\tilde{u}}_{2}-a\int_{0}^{2\ell }{\tilde{T}}_{x}^{2}dx\;-\frac{a}{b}\left(\bar{T}\left(s_{1}\right){\dot{s}}_{1}+T\left({\bar{s}}_{1}\right){\overset{\cdot }{\bar{s}}}_{1}\right)+\frac{a}{b}\left(\bar{T}\left(s_{2}\right){\dot{s}}_{2}+T\left({\bar{s}}_{2}\right){\overset{\cdot }{\bar{s}}}_{2}\right).$$

To complete the proof, we need the bounds on $\tilde{T}$ and ${\tilde{T}}_{x}$ that came from Poincaré’s and Agmon’s inequalities. These relied on knowing that $\tilde{T}(\ell )=0$ due to symmetry, which is not necessarily true anymore.

There are two possible cases. First, the liquid phases of the reference and the actual system overlap at some point, i.e., $T\left(x_{eq}\right)=\overset{‾}{T}\left(x_{eq}\right)=T_{f}$, for some $0<x_{eq}<2\ell$. Second, there is no overlap. This necessarily means that $s_{2}<{\overset{‾}{s}}_{1}$, or $s_{1}>{\overset{‾}{s}}_{2}$. If the former is true, Assumption (A5) implies that $T\left(s_{2}\right)=T_{f}$ and $T\left({\overset{‾}{s}}_{1}\right)<T_{f}$, while $\overset{‾}{T}\left(s_{2}\right)<T_{f}$ and $\overset{‾}{T}\left({\overset{‾}{s}}_{1}\right)=T_{f}$, and the two temperature profiles are continuous. Therefore, they must intersect at some point $s_{2}<x_{eq}<{\overset{‾}{s}}_{1}$. Similar reasoning holds if $s_{1}>{\overset{‾}{s}}_{2}$. Thus, we must have a point where the temperatures are equal, and $\tilde{T}\left(x_{eq}\right)=0$, which allows us to apply Poincaré’s and Agmon’s inequalities.

The remainder of the proof follows that of Theorem 3.1. ∎

*Remark IV.I:* There are other control laws that satisfy (76) that may still provide uniform convergence of $\tilde{T}$, but the convergence could happen after the strand becomes completely solid (that is, the two boundaries meet).

In addition to (75), consider the following control law that satisfies (76):

(80) $${\tilde{u}}_{1}={\tilde{u}}_{2}=\frac{k}{2}\left(\frac{1}{a}\int_{0}^{2\ell }\tilde{T}dx+\frac{1}{b}\left({\tilde{s}}_{2}-{\tilde{s}}_{1}\right)\right)$$

**Fig. 11.**
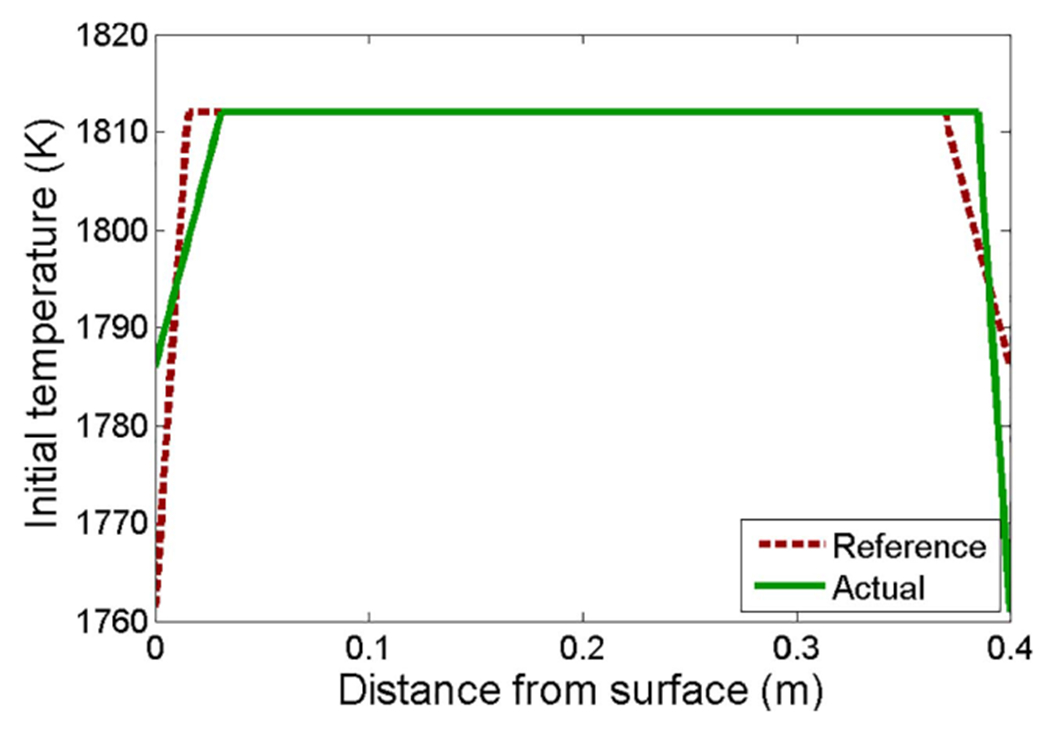
Initial condition for simulation with symmetric enthalpy error.

**Fig. 12.**
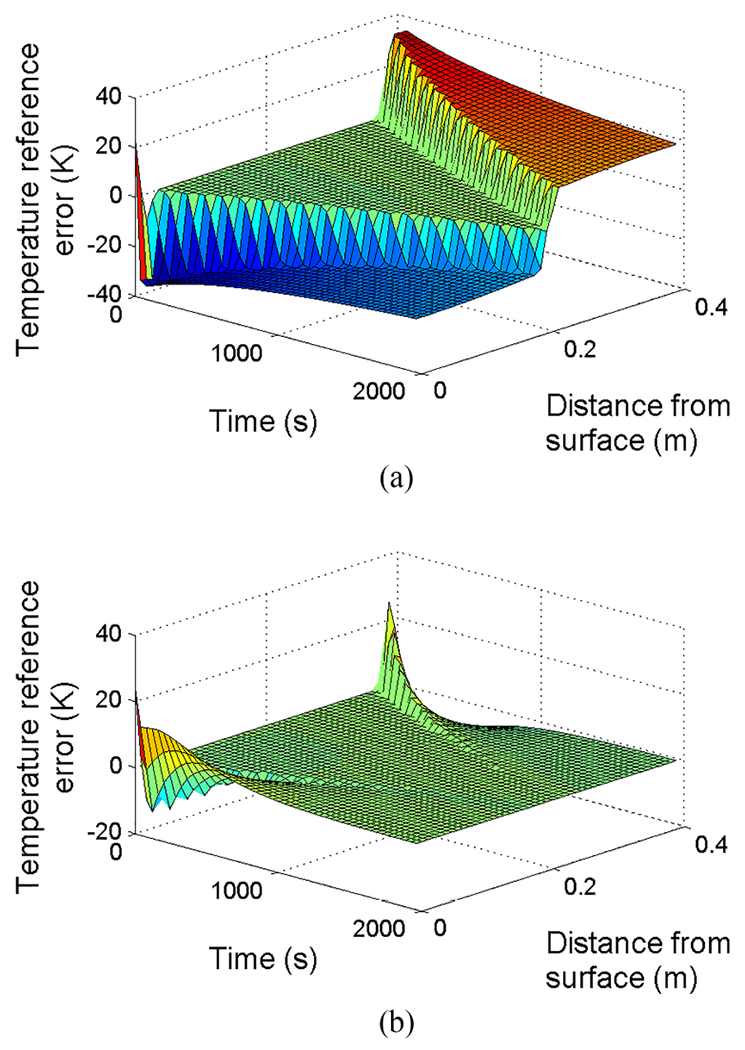
Temperature error $\tilde{T}$ for two-sided Stefan problem (62)–(65), with initial conditions from Fig. 11 and different boundary control. (a) Using Neumann boundary control (80). (b) Using Neumann boundary control (75).

Note that both (75) and (80) satisfy (76). However, suppose the initial condition has an error as shown in Fig. 11. Since the error in enthalpy is symmetric, the control law (80) will not make any adjustments. The errors will eventually converge, but not until after the final solidification, as shown in Fig. 12(a). For the intended application of continuous casting, such a slow convergence will fail to meet the quality and safety goals.

Compare this with the closed-loop performance under control law (75), which is simulated in Fig. 12(b). The closed-loop under the second control law converges at the initial stage of solidification, illustrating the importance of applying the form of the control law which provides the best convergence rate in the two-sided case.

### B. Observer Design

Similarly, we can extend the observer (40)–(43) designed in Section III to the two-sided Stefan problem as follows:

(81) $${\hat{T}}_{t}(x,t)=a{\hat{T}}_{xx}(x,t),\;x\in (0,\ell )\backslash \left\{{\hat{s}}_{1}(t),{\hat{s}}_{2}(t)\right\}$$

(82) $$\hat{T}\left({\hat{s}}_{1}(t),t\right)=T_{f}=\hat{T}\left({\hat{s}}_{2}(t),t\right)\;{\hat{T}}_{x}(0,t)=u_{1}(t),\;{\hat{T}}_{x}(2\ell ,t)=u_{2}(t),\;{\hat{T}}_{x}(\ell ,t)=0$$

(83) $$\hat{T}(x,0)={\hat{T}}_{0}(x),\;{\hat{s}}_{1}(0)={\hat{s}}_{1,0},\;{\hat{s}}_{2}(0)={\hat{s}}_{2,0}$$

(84) $${\overset{\cdot }{\hat{s}}}_{1}(t)=-b\left({\hat{T}}_{x}\left({\hat{s}}_{1}^{+}(t)\right)-{\hat{T}}_{x}\left({\hat{s}}_{1}^{-}(t)\right)\right)\;+l(\hat{T}(0,t)-T(0,t))\;{\overset{\cdot }{\hat{s}}}_{2}(t)=-b\left({\hat{T}}_{x}\left({\hat{s}}_{1}^{+}(t)\right)-{\hat{T}}_{x}\left({\hat{s}}_{2}^{-}(t)\right)\right)\;-l(\hat{T}(2\ell ,t)-T(2\ell ,t))$$

where the initial conditions satisfy Assumption (A5) and the estimation gain l>0. Then, we can extend Theorem 3.1 as follows.

*Theorem IV.2:* Consider the observer system (81)–(84). Suppose the initial conditions satisfy assumptions (A5) and (A2), and the boundary flux satisfies assumption (A3). Then with a sufficiently small estimation gain $l,\overset{ˆ}{T}$ converges asymptotically to T uniformly over the domain, and ${\overset{ˆ}{s}}_{1}$ and ${\overset{ˆ}{s}}_{2}$ asymptotically and monotonically converge, respectively, to $s_{1}$ and $s_{2}$.

*Proof:* The proof follows the same basic principle as the proof of Theorem 3.1. Consider the following Lyapunov functional candidate:

(85) $$V(\hat{T}-T):=\frac{1}{2}\int_{0}^{2\ell }{\left(\hat{T}-T\right)}^{2}\text{dx}-\frac{a}{b}T_{f}\left(s_{1}+{\hat{s}}_{1}\right)+\frac{a}{b}T_{f}\left(s_{2}+{\hat{s}}_{2}\right).$$

Taking the time derivative of (85) yields

(86) $$\dot{V}=-a\int_{0}^{2\ell }{\left({\hat{T}}_{x}-T_{x}\right)}^{2}dx-\frac{a}{b}\left(T\left({\hat{s}}_{1}\right){\overset{\cdot }{\hat{s}}}_{1}+\hat{T}\left(s_{1}\right){\dot{s}}_{1}\right)\;+\frac{a}{b}\left(T\left({\hat{s}}_{2}\right){\overset{\cdot }{\hat{s}}}_{2}+\hat{T}\left(s_{2}\right){\dot{s}}_{2}\right)\;-\frac{l}{b}\left(\hat{T}\left({\hat{s}}_{1}\right)-T\left({\hat{s}}_{1}\right)\right)(\hat{T}(0)-T(0))\;-\frac{l}{b}\left(\hat{T}\left({\hat{s}}_{2}\right)-T\left({\hat{s}}_{2}\right)\right)(\hat{T}(2\ell )-T(2\ell )).$$

To complete the proof, we need the bounds on $\overset{ˆ}{T},T,T_{x}$, and ${\overset{ˆ}{T}}_{x}$ that came from Pointcaré’s and Agmon’s inequalities. Similarly, as in Theorem 4.1, the existence of bounds can be derived for $\overset{ˆ}{T},T,T_{x}$, and ${\overset{ˆ}{T}}_{x}$. Then, choosing

(87) $$l\leq \text{min}\left(\frac{b{\text{min}}_{t}\left|{\hat{T}}_{x}\left({\hat{s}}_{1}^{-}\right)\right|}{{\text{max}}_{t}|\hat{T}(0)-T(0)|},\frac{b{\text{min}}_{t}\left|{\hat{T}}_{x}\left({\hat{s}}_{2}^{+}\right)\right|}{{\text{max}}_{t}|\hat{T}(2\ell )-T(2\ell )|}\right)$$

ensures that ${\dot{\overset{ˆ}{s}}}_{1}\geq 0,\;{\dot{\overset{ˆ}{s}}}_{2}\leq 0$. Choosing an appropriate temperature scale to ensure $\overset{ˆ}{T}(s)$ and $T(\overset{ˆ}{s})$ are non-negative yields

(88) $$\dot{V}\leq -a\overset{2\ell }{\underset{0}{\int }}{\left({\hat{T}}_{x}-T_{x}\right)}^{2}dx\;-\frac{l}{b}\left(\hat{T}\left({\hat{s}}_{1}\right)-T\left({\hat{s}}_{1}\right)\right)(\hat{T}(0)-T(0))\;-\frac{l}{b}\left(\hat{T}\left({\hat{s}}_{2}\right)-T\left({\hat{s}}_{2}\right)\right)(\hat{T}(2\ell )-T(2\ell )).$$

Then, applying Pointcaré’s inequality, yields

(89) $$\dot{V}\leq -\frac{a}{16\ell^{2}}\int_{0}^{2\ell }{\left(\hat{T}-T\right)}^{2}dx\;-\frac{l}{b}\left(\hat{T}\left({\hat{s}}_{1}\right)-T\left({\hat{s}}_{1}\right)\right)(\hat{T}(0)-T(0))\;\;-\frac{l}{b}\left(\hat{T}\left({\hat{s}}_{2}\right)-T\left({\hat{s}}_{2}\right)\right)(\hat{T}(2\ell )-T(2\ell )).$$

The remainder of the proof follows that of Theorem 3.1. ∎

### C. Output Feedback Controller Design

*Theorem IV.3:* Suppose $\left(T,s),(\overset{‾}{T},\overset{‾}{s}\right)$, and $\left(\overset{ˆ}{T},\overset{ˆ}{s}\right)$ all satisfy the hypotheses of Theorem 3.1. Then, under the control law

(90) $${\tilde{u}}_{1}={\bar{u}}_{1}+K\left(\frac{1}{a}\int_{0}^{\ell }(\hat{T}-\bar{T})dx-\frac{1}{b}\left({\hat{s}}_{1}-{\bar{s}}_{1}\right)\right)\;{\tilde{u}}_{2}={\bar{u}}_{2}+K\left(\frac{1}{a}\int_{\ell }^{2\ell }(\hat{T}-\bar{T})dx+\frac{1}{b}\left({\hat{s}}_{2}-{\bar{s}}_{2}\right)\right)$$

where gain K>0, the temperature reference error $\tilde{T}$ and the estimation error $\overset{ˆ}{T}-T$ uniformly converge to 0, and $s_{1}$ and $s_{2}$ asymptotically and monotonically converge, respectively, to ${\overset{‾}{s}}_{1}$ and ${\overset{‾}{s}}_{2}$.

*Proof:* Denote ${\tilde{u}}_{1}=u_{1}-{\overset{‾}{u}}_{1},{\tilde{u}}_{2}=u_{2}-{\overset{‾}{u}}_{2}$, the control law can be rewritten as

(91) $${\tilde{u}}_{1}+{\tilde{u}}_{2}=K(\hat{H}-\bar{H}).$$

Taking its time derivative yields

(92) $${\overset{\cdot }{\tilde{u}}}_{1}+{\overset{\cdot }{\tilde{u}}}_{2}=-K\left({\tilde{u}}_{1}+{\tilde{u}}_{2}\right)-\frac{Kl}{b}(\hat{T}(0)-T(0))\;-\frac{Kl}{b}(\hat{T}(2\ell )-T(2\ell ))$$

which implies that ${\tilde{u}}_{1}+{\tilde{u}}_{2}$ converges to 0 as $\tilde{T}$ converges asymptotically to T uniformly over the domain.

Consider now the following Lyapunov functional candidate:

(93) $$V(t):=\frac{1}{2}\int_{0}^{2\ell }{\tilde{T}}^{2}dx+\frac{1}{2}\int_{0}^{2\ell }{\left(\hat{T}-T\right)}^{2}dx\;-\frac{a}{b}T_{f}\left({\hat{s}}_{1}+2s_{1}+{\bar{s}}_{1}\right)+\frac{a}{b}T_{f}\left({\hat{s}}_{2}+2s_{2}+{\bar{s}}_{2}\right).$$

Taking its time derivative yields

(94) $$\dot{V}=-a\tilde{T}(0){\tilde{u}}_{1}-a\tilde{T}(2\ell ){\tilde{u}}_{2}\;-a\int_{0}^{2\ell }{\tilde{T}}_{x}^{2}dx-a\int_{0}^{2\ell }{\left({\hat{T}}_{x}-T_{x}\right)}^{2}dx\;-\frac{a}{b}\left(\bar{T}\left(s_{1}\right){\dot{s}}_{1}+T\left({\bar{s}}_{1}\right){\dot{s}}_{1}\right)+\frac{a}{b}\left(T\left({\hat{s}}_{2}\right){\overset{\cdot }{\hat{s}}}_{2}+\hat{T}\left(s_{2}\right){\dot{s}}_{2}\right)\;-\frac{a}{b}\left(T\left({\hat{s}}_{1}\right){\overset{\cdot }{\hat{s}}}_{1}+\hat{T}\left(s_{1}\right){\dot{s}}_{1}\right)+\frac{a}{b}\left(T\left({\hat{s}}_{2}\right){\overset{\cdot }{\hat{s}}}_{2}+\hat{T}\left(s_{2}\right){\dot{s}}_{2}\right)\;\;-\frac{l}{b}\left(\hat{T}\left({\hat{s}}_{1}\right)-T\left({\hat{s}}_{1}\right)\right)(\hat{T}(0)-T(0))\;-\frac{l}{b}\left(\hat{T}\left({\hat{s}}_{2}\right)-T\left({\hat{s}}_{2}\right)\right)(\hat{T}(2\ell )-T(2\ell ))\;\leq -\frac{a}{16\ell^{2}}\left(\int_{0}^{2\ell }{\tilde{T}}^{2}dx+\int_{0}^{2\ell }{\left(\hat{T}-T\right)}^{2}dx\right)\;-\frac{l}{b}\left(\hat{T}\left({\hat{s}}_{1}\right)-T\left({\hat{s}}_{1}\right)\right)(\hat{T}(0)-T(0))\;-\frac{l}{b}\left(\hat{T}\left({\hat{s}}_{2}\right)-T\left({\hat{s}}_{2}\right)\right)(\hat{T}(2\ell )-T(2\ell )).$$

Following the discussion similar to the proof of Theorem 3.1 yields

(95) $$\dot{V}\leq -\frac{a}{16\ell^{2}}\left(\int_{0}^{\ell }{\tilde{T}}^{2}dx+\int_{0}^{\ell }{\left(\hat{T}-T\right)}^{2}dx\right).$$

Applying the invariance principle and the interface convergence arguments finishes the proof. ∎

### D. Controller Implementation and Numerical Error Effects

The practical implementation of the control laws presented requires their finite-dimensionalization. Rigorous formal error bounds assessment for the latter is nontrivial due to system’s hard nonlinearity (enthalpy jump), and could be addressed through nonsmooth analysis tools, such as partial differential inequalities and Clarke’s proximal superdifferentials, proven useful in the ${𝒣}_{\infty }$ design for systems with hard nonlinearities in [45]. This, however, is likely to yield conservative error bounds. The diffusive nature of the operators involved and the ensuing prevalent solution monotonicity make this assessment not as critical, so that heuristic numerical assessment of the error effects could yield a reasonable degree of confidence in the implemented controller performance. In [35], Ch. 11] such a numerical closed loop approximation of an output feedback controller consisting of a PDE observer and a full-state feedback SITE/IPE control law is carried out and implemented in real-time in the experimental study of paraffin melting control. In [35], Sec. 11.5], the closed-loop performance is presented matching the theoretically expected result of the fixed interface position setpoint control and in this sense, makes an informal assessment of the error bounds. In the application targeted by this article—steel casting—a similar assessment has been carried out in the actual industrial application [22], where a real-time control system, CONONLINE, is described.

The system features an efficient fundamentally based solidification heat-transfer model, CONSENSOR that estimates the entire shell surface temperature and solidification profile in real time, based on tracking multiple horizontal slices through the strand with a subroutine version of a 1-D computational model (CON1D). CON1D uses a generalized form of conservation energy to model the 1-D slice equivalent to the Stefan model in a weak sense [41]. In the application papers [2], [46], 200 and 500 “CON1D” slices are simulated in real time.

Numerically, CON1D employs a finite-difference scheme that is the second-order accurate in the space step size and the first-order accurate in the time step size. When the grid spacing becomes small enough, the floating point error begins to dominate the finite-difference error. Balancing of the computational tradeoffs produced negligible real-time computational error.

**Fig. 13.**
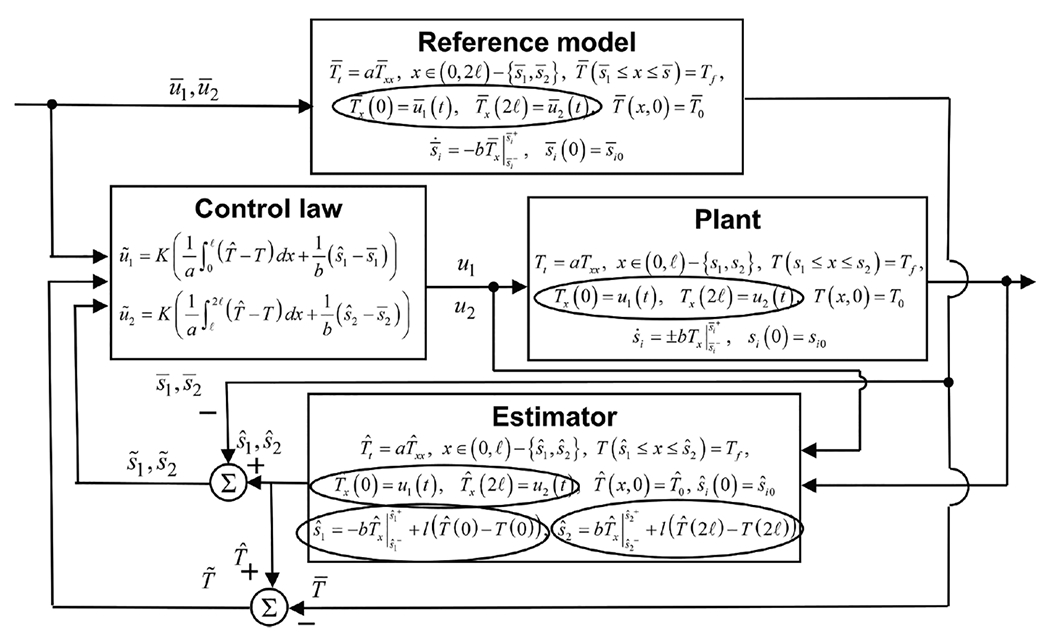
Block diagram for the proposed two-sided Stefan problem control law (90).

The block diagram of the two-sided output-feedback system with control law (90) shown in Fig. 13 provides a practical framework to embed the control algorithms designed in this article into CONONLINE implemented on two casters at Nucor Steel Decatur. CONSENSOR has been operating on two continuous casters at Nucor Decatur for more than 10 years, and has been used by plant engineers and operators to monitor the process. Successful trials with the CONONLINE in control of the temperature have also been run, however, the feedback control part requires an upgrade before it could be put into regular production. This article provides a step toward such an upgrade.

## V. Conclusion

In this article, output feedback control of the Stefan problem through the solid boundary is proposed using the enthalpy approach. The closed-loop stability and the temperature and solidification front error convergence in tracking the reference model trajectory are shown through a Lyapunov functional. The control law for the two-sided Stefan problem arising in most applications is also presented. Also, through simulation, exponential error convergence rate is demonstrated. The results presented bridge the gap between the needs of the continuous steel casting and the available control techniques and also provide guidance for caster sensing/actuation upgrades when higher process performance is desired.

Future work includes developing the techniques for the agile reference model updates in response to the range of production changes, as well as the control laws modification to meet the practical constraints on sensing and actuation.

## Figures and Tables

**TABLE I:** Thermodynamic Properties Used in Simulations

Symbol	Description	Value
k	thermal conductivity	80.6W/m·K
$c_{p}$	specific heat	460J/kg·K
$T_{f}$	melting temperature	1580°C
ℓ	half-thickness of strand	0.2m
ρ	density	$7.87\times 10^{3}\;kg/m^{3}$
$\overset{‾}{u}$	constant reference control	3000K/m
